# c-Fos induces chondrogenic tumor formation in immortalized human mesenchymal progenitor cells

**DOI:** 10.1038/s41598-018-33689-0

**Published:** 2018-10-23

**Authors:** Ander Abarrategi, Stefano Gambera, Arantzazu Alfranca, Miguel A. Rodriguez-Milla, Raquel Perez-Tavarez, Kevin Rouault-Pierre, Alexander Waclawiczek, Probir Chakravarty, Francisca Mulero, César Trigueros, Samuel Navarro, Dominique Bonnet, Javier García-Castro

**Affiliations:** 1 Unidad de Biotecnología Celular, Instituto de Salud Carlos III, Madrid, E-28021 Spain; 2 Haematopoietic Stem Cell Laboratory, The Francis Crick Institute, London, WC2A 3LY UK; 3 Unidad de Histología, Instituto de Salud Carlos III, Madrid, E-28021 Spain; 4 Bioinformatics Core, The Francis Crick Institute, London, United Kingdom; 5 Molecular Image Core Unit, Spanish National Cancer Research Centre, Madrid, E-28029 Spain; 6 Mesenchymal and Hematopoietic Stem Cell Laboratory, Fundación Inbiomed, San Sebastian, E-20009 Spain; 7 Pathology Department, University of Valencia, Valencia, E-46010 Spain

## Abstract

Mesenchymal progenitor cells (MPCs) have been hypothesized as cells of origin for sarcomas, and c-Fos transcription factor has been showed to act as an oncogene in bone tumors. In this study, we show c-Fos is present in most sarcomas with chondral phenotype, while multiple other genes are related to c-Fos expression pattern. To further define the role of c-Fos in sarcomagenesis, we expressed it in primary human MPCs (hMPCs), immortalized hMPCs and transformed murine MPCs (mMPCs). In immortalized hMPCs, c-Fos expression generated morphological changes, reduced mobility capacity and impaired adipogenic- and osteogenic-differentiation potentials. Remarkably, immortalized hMPCs or mMPCs expressing c-Fos generated tumors harboring a chondrogenic phenotype and morphology. Thus, here we show that c-Fos protein has a key role in sarcomas and that c-Fos expression in immortalized MPCs yields cell transformation and chondrogenic tumor formation.

## Introduction

Osteosarcomas (OS) and chondrosarcomas (CS) are the most prevalent primary bone tumors. The identity of cells of origin of those tumors is certainly controversial^1–7^ and therefore better understanding of the cellular origin of these tumors is needed to improve patient outcome^1^. There is increasing evidence showing that mesenchymal progenitor cells (MPCs) may act as cells of origin of sarcomas. Murine MPCs (mMPCs) with mutations in p21, p53 and/or Rb serve as cell of origin of fibrosarcoma, leiomyosarcoma and OS^8–10^. Likewise, overexpression of c-MYC in p16INK4A−/−p19ARF−/− murine MPCs results in OS development^11^. Human MPCs (hMPCs) are more resistant to tumoral transformation, and therefore several events need to be combined to achieve an oncogenic phenotype, such as introduction of human telomerase (TERT), expression of HPV-16 E6 and E7 to abrogate the functions of p53 and pRB family members, expression of SV40 small T or large T antigens to inactivate protein phosphatase 2A (PP2A) and therefore stabilize c-Myc, and finally induction of H-RAS, a well-known oncogene^12–14^. These transformed hMPCs generate *in vivo* tumors classified as undifferentiated spindle cell sarcomas.

In the case of CS, the cell of origin for peripheral chondrosarcoma seems to arise from differentiated chondrocytes. In example, Osteochondroma appears when Ext1 is inactivated in the growth plate’s chondrocyte^15^ and p53/p16 inactivated in these mice^16^. In the case of central chondrosarcomas, mutations in IDH push MPCs towards chondrogenic differentiation instead of osteogenic differentiation causing enchondromas, and additional mutations are required for progression towards chondrosarcoma^17^. However, different progenitors maybe involved in CS formation, as hierarchical clustering of MPCs gene expression during chondrogenesis allowed the classification of patient-samples in clusters corresponding to the phenotypes of chondrosarcoma in early and late differentiation stage^18^.

AP-1 is a transcription complex composed by members of the Jun, Fos, and activating transcription factor (ATF) family of proteins that bind as hetero- and/or homodimers to AP-1 binding sites in the promoters of various target genes. c-Fos is expressed during early bone differentiation^5,19^, and plays a crucial role in regulating endochondral osteogenesis in bone formation and fracture healing^20,21^. *In vitro*, cartilage progenitor cells of mouse mandibular condyles differentiate to osteoblasts, and generate temporal pattern of c-Fos expression in hypertrophic chondrocytes, which precedes the expression of bone cell characteristic genes^22^.

Mice and human OS are frequently characterized by high c-Fos expression^23,24^. In transgenic mice models, the induction of c-Fos expression using ubiquitous promoter results in specific transformation of osteoblasts, leading to OS formation^5^; on the contrary, chimeric mice obtained from c-Fos-overexpressing embryonic stem cells develop chondrogenic tumors^25^. Overexpression of c-Fos in already transformed p53 deficient mouse MPCs yields a shift from osteoblastic to chondroblastic phenotype in generated tumors, suggesting a role of c-Fos in defining the tumor phenotype, but these assays show no evidence of the role of c-Fos in cell-transformation process^22,26^. In patients context, some authors speculate that c-Fos and c-Jun may be implicated in the development of high-grade OS, as they do not detect significant expression of these factors in low-grade OS and cartilaginous skeletal neoplasms^27^. By contrast, in other study c-Fos expression was detected in 50% human CS tumors^28^, while inoculation of human CS HCS-2/8 cells in nude mice generates CS with high expression levels of c-Fos^29^.

To sum up, MPCs have been proposed before as cells of origin for OS and CS, but the precise role of c-Fos in human MPCs transformation has not been delineated yet. Here we show that the expression of c-Fos leads to oncogenic transformation of immortalized hMPCs. These c-Fos-transformed hMPCs display reduced adipogenic and osteogenic differentiation potential *in vitro*, with a conserved ability to specifically differentiate into chondrogenic lineage. In accordance, c-Fos-transformed hMPCs give rise to chondrogenic tumors upon implantation in immunodeficient mice. Importantly, most human CS expresses detectable levels of c-Fos protein in tested clinical samples.

## Results

### c-Fos expression pattern is related to multiple cell functions in human sarcomas, while it is especially expressed in those with chondroblastic phenotype

**Figure 1:**
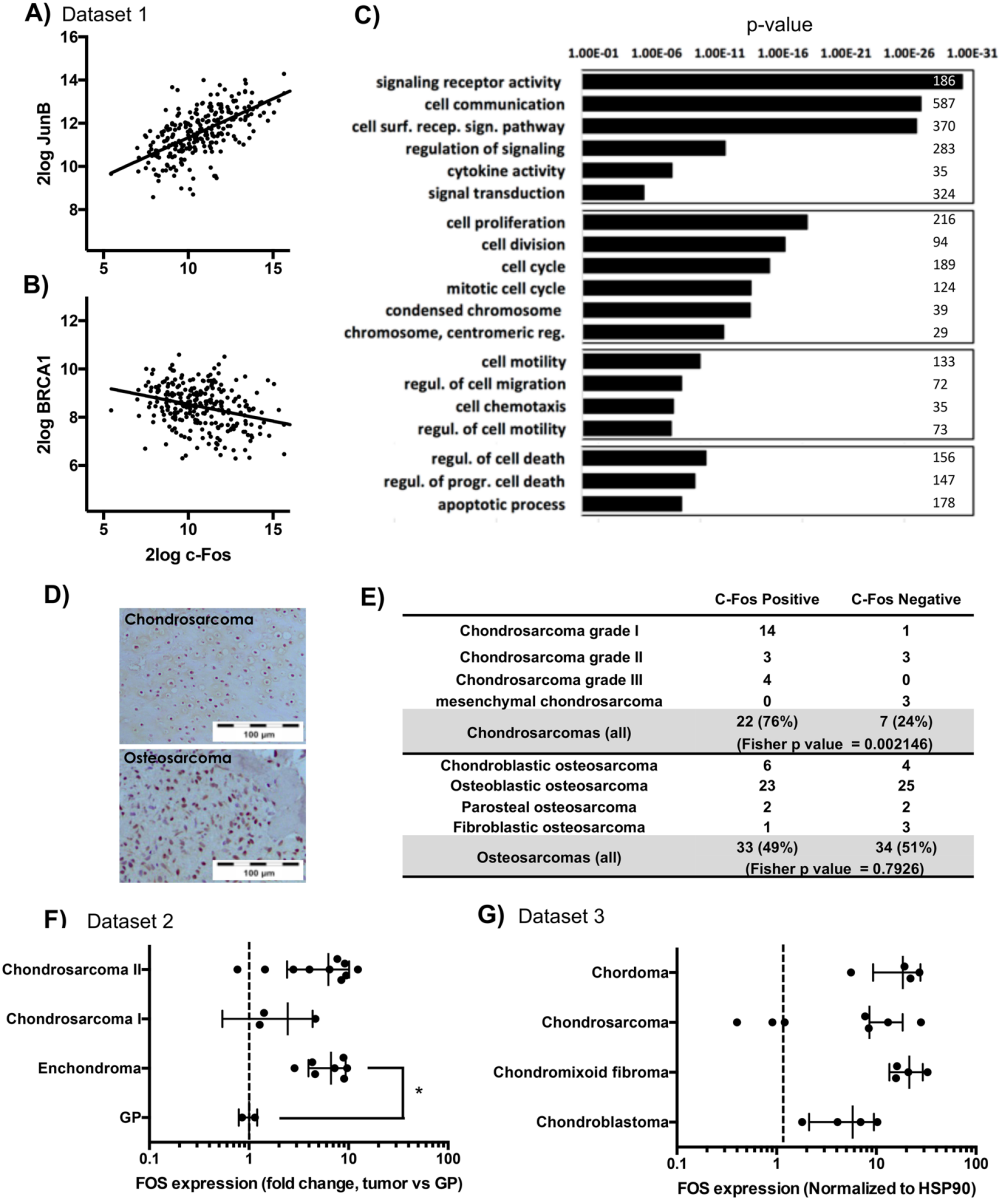
c-Fos expression in human sarcoma samples. (**A**–**C**) Co-expression studies of FOS with other genes in a cohort with 259 sarcoma samples. (See Dataset 1 in Supplementary Materials and methods). (**A**,**B** Pearson correlation study, p < 0.01). (**A**) Example of positive correlation between FOS and JUNB gene expression. Each dot represents a sarcoma sample. (**B**) Example of negative correlation between FOS and BRCA1 gene expression. Each dot represents a sarcoma sample. (**C**) Gene ontology (GO) study of FOS correlated genes (chi-square with continuity correction study, p < 0.01). A list of significant GO terms with the p-values is provided. The number on the right of each GO term represent the number of genes observed for that GO term. (**D**) Representative images of performed c-Fos IHC in clinical samples of human sarcomas (Chondrosarcoma n = 29, Osteosarcoma n = 67). (**E**) Table summarizing the presence of c-Fos in clinical samples of human sarcomas (Fisher’s exact test, p < 0.01). (**F**,**G**) c-Fos expression at mRNA level in different sarcoma tumors (*p ≤ 0.1). (**F**) data extracted from dataset 2, a cohort with 21 sarcoma samples, and normalized to c-Fos expression in growth plate (GP) controls included in the same dataset. (**G**) data from 19 sarcoma samples extracted from dataset 3 and normalized to HSP90 expression in each patient.

We used human sarcoma gene expression datasets in order to define genes following similar (Fig. 1A) or opposite (Fig. 1B) expression patterns than c-Fos. There were 858 genes positively correlated (p < 0.01) and 240 genes negatively correlated with FOS (p < 0.01). We used those genes to define pathways and cellular processes related to them (Fig. 1C), and found many of these genes are significantly related to processes like cell communication, cell proliferation, cell mobility and regulation of cell death, among others (Fig. 1C), which are key cellular processes in tumorogenesis and tumor progression events.

In a more classical approach, we analyzed c-Fos protein expression by immunohistochemistry in tissue microarrays of human bone sarcomas, and we detected this protein in 76% of human CS (n = 29) and 49% of human OS (n = 67) (Fig. 1D,E). Interestingly, c-Fos expressed in CS samples showed a predominant nuclear staining, suggesting its participation in active transcription processes (Fig. 1D). We examined c-Fos mRNA expression in human sarcoma samples and found c-Fos is also expressed at mRNA level in different types of sarcomas with chondral phenotype (Fig. 1F,G), which is in consonance with the immunohistochemistry data.

### c-Fos expression in immortalized human MPCs promotes cell transformation

In order to define the role of c-Fos on hMPCs, we initially transduced bone marrow-derived primary hMPCs with a lentiviral vector designed for c-Fos expression. We observed a higher proliferation rate in c-Fos-expressing hMPCs compared to empty lentiviral vector transduced control cells, but not immortalization or transformation of primary hMPCs. A β-gal assay confirmed senescence in transduced cells after mid-term culture (Supplementary Fig. 1a,b).

**Figure 2:**
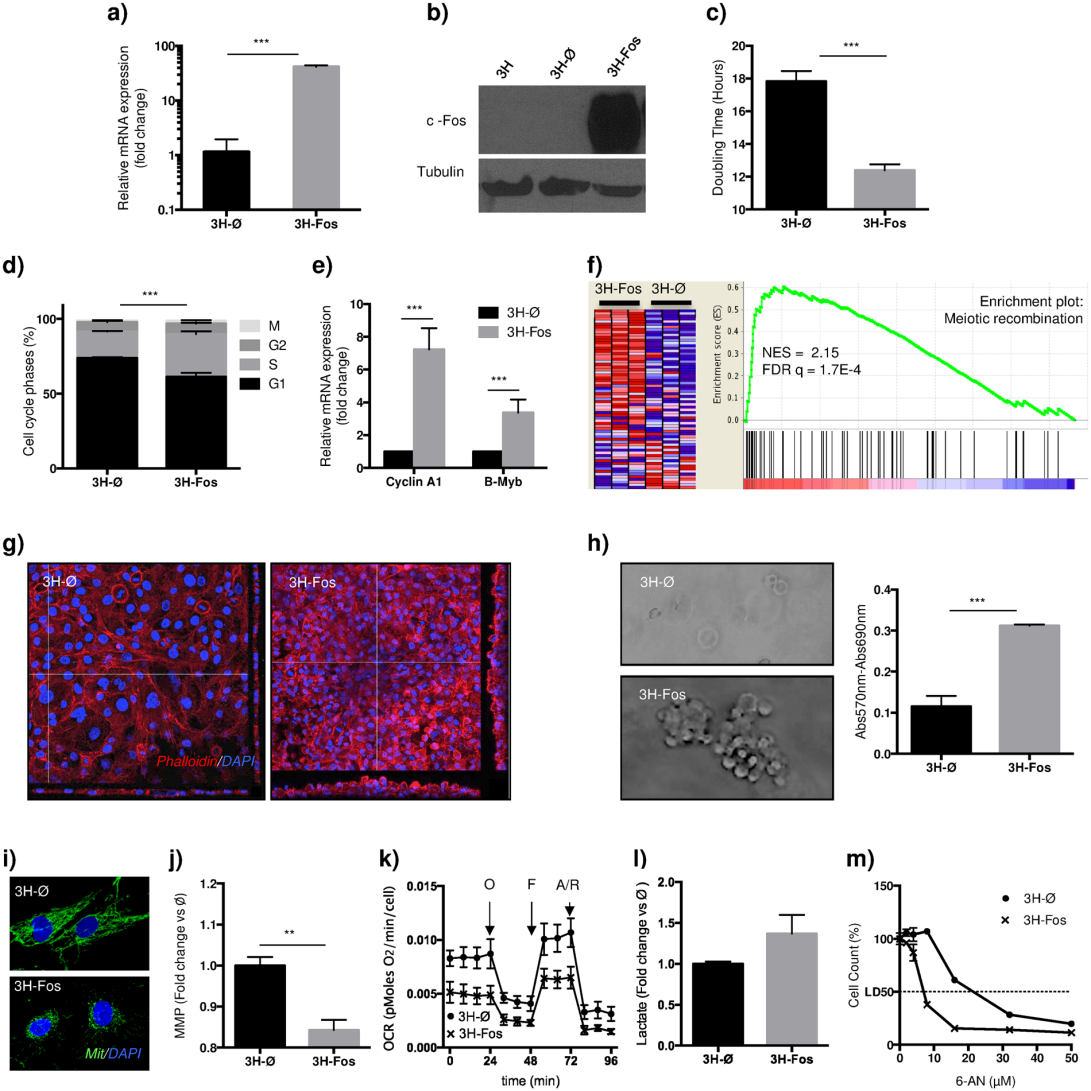
c-Fos expression induces 3H cells transformation. **(a**) RT-qPCR of c-Fos expression in transduced cells (n = 3). (**b**) Western blot showing c-Fos expression (n = 3). (**c**) Quantification of cell doubling time (n = 3). (**d**) Cell cycle study showing percentage of cells in each cell cycle phase (n = 3, ANOVA with multiple comparison test). (**e**) RT-qPCR of Cyclin A1 (CCN1) and B-Myb (MYBL2) expression levels (n = 4). (**f**) “Reactome_Meiotic recombination” Specific gene set enriched in 3H-Fos cells. Data obtained from GSEA pathway study. (**g**) Confocal images depicting z-stack reconstruction (Red: Phalloidin staining, Blue: DAPI) (n = 3). (**h**) Representative images of semisolid cultures at day 7, and quantification of MTT reduction by viable cells recovered from cultures (n = 4). (**i**) Immunofluorescence images of mitochondrial network (Green: Mitochondria, Blue: DAPI) (n = 3). (**j**) Quantification of Mitochondrial Membrane Potential (MMP) at basal level (n = 5). (**k**) Real time measurement of OCR during sequential addition of mitochondrial function modulation compounds (O: Oligomycin, F: FCCP, A/R: antimycin A and Rotenone) (n = 5 per each point, multiple t-test). (**l**) Lactate accumulation measurement after 48 hours cell culture (n = 3). (**m**) Cell 6-AN drug dose-response assay (n = 3 per each point). (3H-Ø, empty vector transduced cells; 3H-Fos, c-Fos vector transduced cells). (Unless specified, unpaired t-test. *p ≤ 0.05; **p ≤ 0.01; ***p ≤ 0.001).

In view of these results, and given that primary hMPC transformation in culture seems to require multiple oncogenic events^13^, we selected a previously immortalized hMPCs as a model for human cells studies. Those cells, defined as 3 Hits hMPC (3H), overexpresses hTERT and HPV-16 E6/E7, which extend cell lifespan in culture and abrogate p53 and pRB function respectively^12^, although they are no tumorogenic when inoculated in immunodeficient mice (3H in Table 1). We perform lentiviral transduction of 3H cells with c-Fos expressing (3H-Fos) or empty (3H-Ø) lentiviral vectors and c-Fos expression was assessed at mRNA and protein level (Fig. 2a,b). A panel of cell surface markers usually employed to define hMPCs was also used to assess cell phenotype (Supplementary Fig. 1c).

**Table 1:** Table summarizing data from *in vivo* experiments.

	Subcutaneous Tumors					Orthotopic Tumors					IV	
	Tumor incidence	Latency (days)	S100	Alcian blue	Col2	Tumor incidence	Latency (days)	S100	Alcian blue	Col2	Tumor incidence	Latency (days)
3H	0/6					N/A					N/A	
3H-Ø	0/10					0/10					N/A	
3H-FOS	20/20	71 (56–157)	+	+	+	13/13	84 (70–100)	+	+	+	0/10	
3H-FOS (2^ND^)	8/8	58 (49–76)	+	+	+	3/3	73 (60–85)	+	+	+	1/5	200
mMPC- Ø	N/A					4/6	72 (47–91)	−	−	+	N/A	
mMPC-FOS	3/3	33 (29–36)	+	+	−	6/6	42 (40–45)	+	+	+	N/A

In accordance to our results in primary hMPCs, we observed a significant increase in cell proliferation upon c-Fos expression in 3H-Fos cells (Fig. 2c). Likewise, cell cycle studies (Fig. 2d,e) revealed an increment in the percentage of 3H-Fos cells undergoing S and M phases, as compared to 3H-Ø cells (Fig. 2d and Supplementary Fig. 1d). Accordingly, levels of B-Myb and Cyclin A1, which regulate cell cycle progression through G1/S and S/G2/M phases respectively, were also increased in 3H-Fos cells (Fig. 2e and Supplementary Fig. 1e). Gene expression studies were also conducted and they yield gene ontology terms and processes related to cell cycle, as well as other cell processes altered in 3H-Fos cells (Fig. 2f and Supplementary Fig. 2a–d).

We next analyzed whether c-Fos expression induced cell-transformation and whether transformation-related cell functions were altered. Cell cultures revealed that contact inhibition was lost in 3H-Fos cells, as confluent cell monolayers continued growing and formed dense multilayer foci (Fig. 2g). In addition, 3H-Fos cells, but not 3H-Ø cells, were able to form colonies in semisolid media (Fig. 2h), hence indicating c-Fos expression induced effective transformation of these cells. Among transformation-related cell functions, we analyzed data from our gene expression and found significant alterations in genes related to apoptosis and senescence. That finding was further assessed with functional studies showing 3H-Fos cells were more resistant to cell death induced by hyperthermia stress (Supplementary Fig. 2e–h). On the other hand, we noticed a mitochondrial dysfunction in 3H-Fos cells, a feature often described in cell transformation processes. By immunofluorescence assays we observed a change in distribution of cellular mitochondrial network in 3H-Fos cells (Fig. 2i) and also significant decrease of the total mitochondrial membrane potential (Fig. 2j), which encouraged us to measure possible changes in cellular bioenergetics. Indeed, 3H-Fos cells showed a reduction of basal oxygen consumption and diminished mitochondrial and non-mitochondrial respiration rates, measured by serial treatment of the cells with mitochondrial respiratory chain inhibitors and uncouplers, which affect different mitochondrial functions (Fig. 2k). We did not observe significant changes in extracellular lactate measurements (Fig. 2l), while 3H-Fos were more sensitive to treatment with 6-AN (Fig. 2m), a pentose phosphate pathway inhibitor, thereby suggesting 3H-Fos cell-metabolism is imbalanced to increase this pathway related to the synthesis of nucleotides and nucleic acids, and hence to proliferation.

Interestingly, from the array data we observed notable absence of expression modification in genes related to H-RAS and c-myc signaling pathways and target genes, which are the ones altered in previous studies in order to achieve mesenchymal cell transformation via the expression of SV40 small T antigen and H-RAS in these cells^12^ (Supplementary Fig. 3).

Altogether these data indicate that c-Fos expression increases proliferation rate and S/G2/M accumulation, while promotes *in vitro* transformation of immortalized hMPCs possibly related to an increased resistance to death and to mitochondrial dysfunction.

### c-Fos expression in immortalized human MPCs reduce cellular migratory capacity

**Figure 3:**
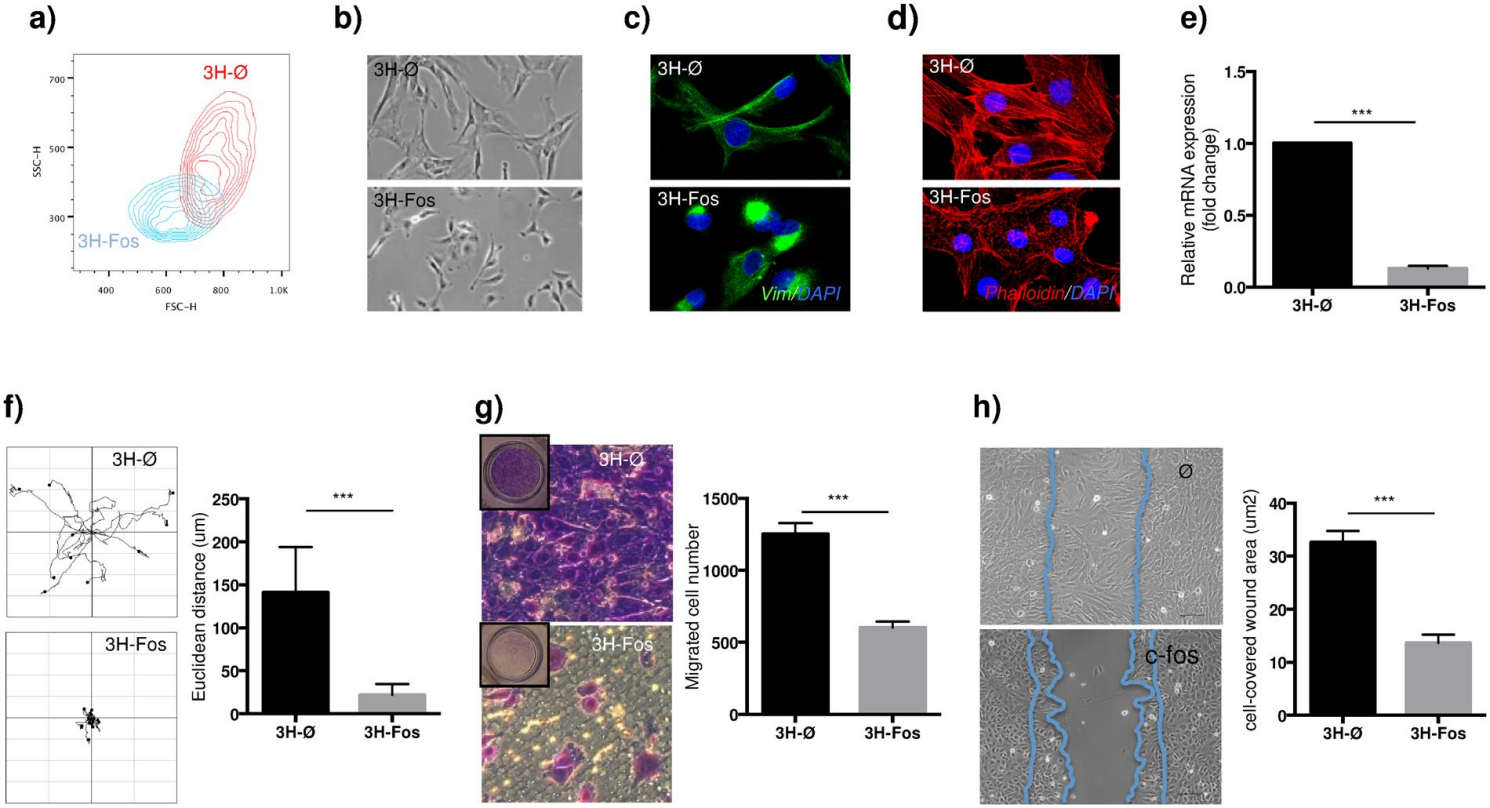
c-Fos induces cytoskeletal changes and suppresses 3H cells invasion properties. (**a**) FACS plot representing cell size and complexity of transduced cells. (**b**) *In vitro* cell morphology after lentiviral transduction. (**c**) Representative immunofluorescence images of vimentin intermediate filament (Green: vimentin, Blue: DAPI) (n = 3). (**d**) Representative immunofluorescence images of Actin cytoskeleton (Red: Palloidin staining, Blue: DAPI) (n = 3). (**e**) RT-qPCR showing Tropomyosin 1 expression (n = 3). (**f**) Graphical representation and quantitative data of cell-displacement 17 hours after seeding at low density (data provided as mean euclidean distance displacement per cell) 10 cells are shown per condition (n = 10). (**g**) Transwell migrated cell-number quantification and representative images of crystal violet stained cells, after 24 hours of migration induction (n = 3). (**h**) Quantification and representative images of wound healing ability in cultured cells measured 12 hours after would formation (quantification is provided as cell-covered wound area) (n = 3). (3H-Ø, empty vector transduced cells; 3H-Fos, c-Fos vector transduced cells.) (unpaired t-test. *p ≤ 0.05; **p ≤ 0.01; ***p ≤ 0.001).

c-Fos expression induced evident changes in cell morphology, including reduced both cell size and intracellular complexity (Fig. 3a,b). Cytoskeleton is related to cell shape and mechanical properties, and therefore the observed morphological changes in 3H-Fos cells suggested possible alterations in cellular cytoskeleton. In this sense, we observed in 3H-Fos cells changes in cellular distribution of vimentin (Fig. 3c), a clear disassembly of actin stress fibers (Fig. 3d) and downregulation of tropomyosin 1 (Fig. 3e), a structural protein implicated in stabilizing cytoskeleton actin filaments. Actin cytoskeleton is also the main force-generating cellular structure and key in whole-cell migration processes. Therefore, data related to changes in cytoskeletal organization led us to investigate whether these changes in actin cytoskeleton could also modify cell migratory capacity. To test this hypothesis, we first analyzed the rate of random motility of individual cells by time-lapse videomicroscopy and found a markedly decreased cell mobility in 3H-Fos compared to 3H-Ø cells (Fig. 3f and Supplementary Fig. S4). In addition to affecting random cell motility, c-Fos expression significantly inhibited stimuli-directed migration, as confirmed in transwell assays (Fig. 3g). Similarly, wound-healing experiments showed that c-Fos expression clearly impaired wound closure in cell culture monolayers of 3H-Fos cells (Fig. 3h).

These results indicate that c-Fos expression in immortalized hMPCs promote changes in cytoskeleton, mainly affecting the ability of cells to generate functional actin cytoskeleton structures. Accordingly, 3H-Fos cells show changes in cell shape and impeded motility features.

### c-Fos expression in immortalized human MPCs modifies differentiation potential

**Figure 4:**
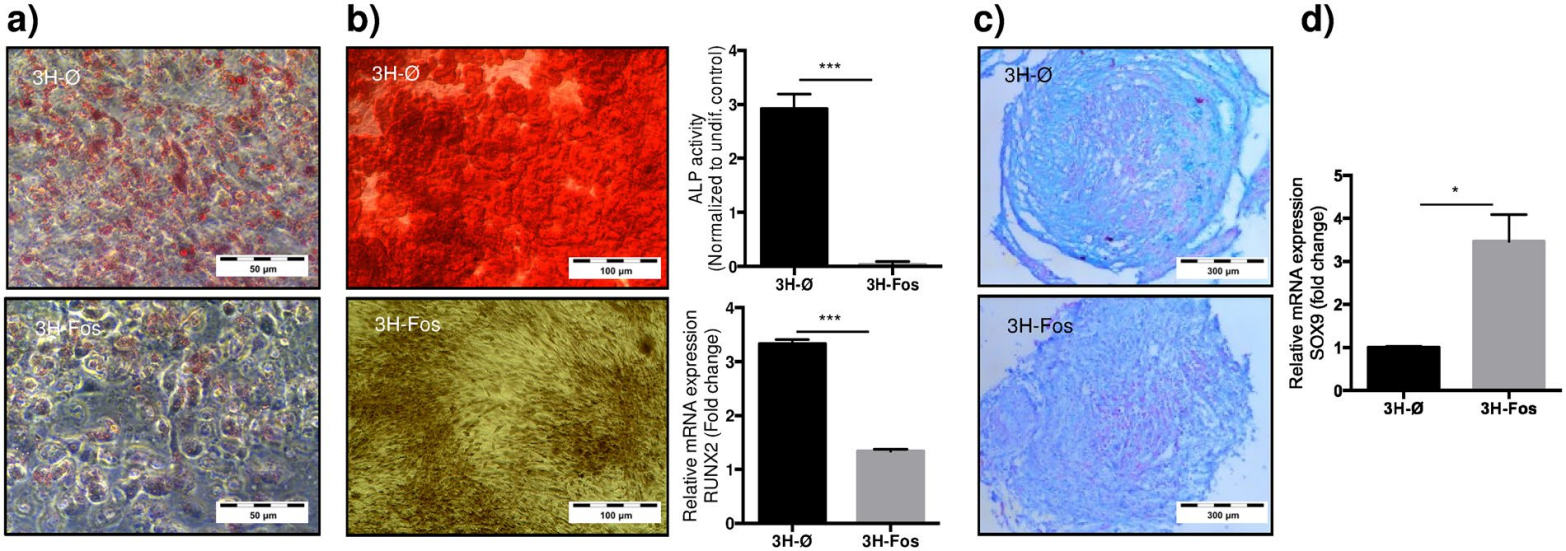
c-Fos induces impaired adipogenic and osteogenic, but not chondrogenic, differentiation in 3H cells. Representative images of (**a**) oil red staining after adipogenic differentiation (n = 5); (**b**) Osteogenic differentiation: alizarin red staining (n = 5), ALP activity (n = 3) and RUNX2 expression (n = 3). ALP protein activity and RT-qPCR of RUNX2 expression were measured 7 days and 24 hours after osteogenic induction, respectively. (**c**) Alcian blue staining after chondrogenesis differentiation in pellet cultures (n = 3). (**d**) Basal SOX9 expression in transduced cells (no differentiation induced) (n = 3). (3H-Ø, empty vector transduced cells; 3H-Fos, c-Fos vector transduced cells) (unpaired t-test. *p ≤ 0.05; **p ≤ 0.01; ***p ≤ 0.001).

In order to define any possible lineage specification related to performed genetic modification, cell differentiation potential studies were performed. In this regard, 3H-Fos cells showed reduced adipogenic and osteogenic differentiation capacity while maintaining chondrogenic differentiation potential, as shown by specific staining techniques (Fig. 4a–c). Accordingly, induction of alkaline phosphatase and RUNX2 mRNA upon osteogenic stimulation was impaired in 3H-Fos comparing with 3H-Ø cells (Fig. 4b), whereas basal expression of SOX9 mRNA, which is related to chondrogenesis, was significantly higher in 3H-Fos cells (Fig. 4d).

These results show that c-Fos expression in immortalized hMPCs induces selective chondrogenic differentiation potential *in vitro*.

### Chondrogenic tumors originate from transformed human MPCs expressing c-Fos

**Figure 5:**
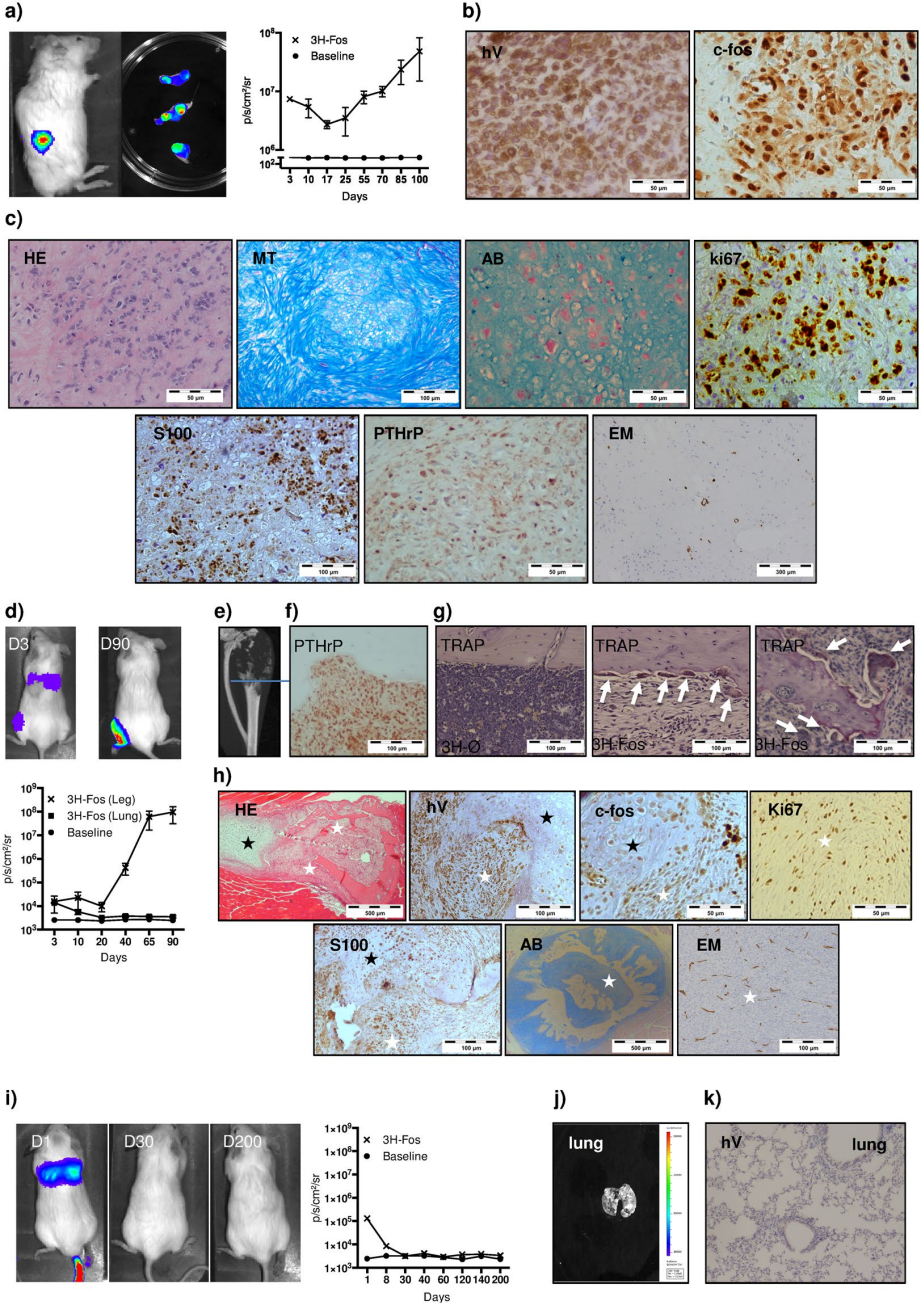
3H-Fos cells generate chondrogenic tumors in immunodeficient mice. (**a**–**c**) Subcutaneous model (n = 20). (**a**) Bioluminiscence images show tumor generation in mice, bioluminescence of extracted tumors and tumor formation bioluminiscence signal-kinetic in mice (n = 3). (**b**) IHC of tumors showing human vimentin and c-Fos expression (note c-Fos is present at nuclear and cytoplasmic level). (**c**) Subcutaneous tumor characterization. Representative images of performed staining and IHC studies are provided. (**d**–**h**) Orthotopic model (n = 13). (**d**) Assessment of tumor formation by bioluminiscence after intra-bone marrow inoculation of 3H-Fos cells (D3, day 3; D90, day 90) (n = 3). (**e**) MicroCT image shows lytic lesion in mouse tibia implanted with 3H-Fos cells (n = 10). (**f**) IHC image shows PTHrP expression in tumoral cells. (**g**) TRAP staining shows osteoclasts (white arrows) in the bone lining area and in the proximity of reactive bone formed in the tumor. (**h**) Representative images of performed staining and IHC studies are provided (black stars indicate point of neoformed cartilage tissue; white starts indicate the location of osteolytic lesions and tumoral cells). (**i**–**k**) Intravenous (i.v.) model (n = 10). (**i**) Bioluminiscence signal-kinetic study after IV inoculation of 3H-Fos cells (D1, day 1; D30, day 30; D200, day 200) (n = 3). (**j**) Lungs did not present bioluminiscence signal at experimental end point. (**k**) No micrometastasis were observed in histological sections (hV, Vimentin; H/E, hematoxilin and eosin; MT, Masson’s trichromic; AB, Alcian Blue; EM, endomucin).

Given that c-Fos expression in immortalized hMPCs promoted increased cell proliferation and transformation *in vitro*, we analyzed whether c-Fos also conferred *in vivo* tumorigenic capacity to these cells. To this end, we conducted xenograft experiments in immunodeficient NOD/SCID mice. Cells of interest were transduced with a constitutive luciferase-expressing lentiviral vector in order to monitor tumor development by bioluminescence, and then implanted subcutaneously (Fig. 5a–c). While control cells did not form tumors, 3H-Fos cells gave rise to rapidly developing subcutaneous tumors composed by highly proliferating cells, as assessed by bioluminescence techniques and further histological ki-67 expression studies (Fig. 5a,c). Human origin of tumoral cells was determined by analysis of human-specific vimentin immunostaining and c-Fos expression of tumor cells (Fig. 5b). Chondrogenic nature of these tumors was further confirmed by histological techniques and immunohistochemistry with specific antibodies (Fig. 5c). In accordance with *in vitro* differentiation assays, tumor was classified as a neoplasia of chondrogenic lineage with scant vascularization, although sparse areas of osteoid could also be found (Supplementary Fig. S5a). Cells from some of these tumors were harvested and re-implanted subcutaneously in secondary recipient mice, yielding tumors with faster formation kinetics and the same histological phenotype as the original ones (Table 1).

Tumor phenotype can be modulated by signals present in host microenvironment^30^, so we carried out orthotopic intratibial implantation of 3H-Fos cells and followed up tumor development by bioluminescence (Fig. 5d). MicroCT images demonstrated the presence of osteolytic lesion of neoplasic appearance (Fig. 5e). Interestingly, neoplastic cells also expressed parathyroid hormone-related protein (PTHrP), a hormone involved in osteoclastogenesis (Fig. 5f), in agreement with the presence of TRAP positive osteoclasts close to both native and reactive bone line (Fig. 5g). Again, tumor cells expressed specific chondral markers, while areas of neoplastic osteoid formation could also be observed (Fig. 5h and Supplementary Fig. S5b). Endomucin staining indicated an increased vascular density in orthotopic tumors, whose human origin was confirmed by human vimentin and c-Fos expression (Fig. 5h). Similar to subcutaneous model, in re-implantation studies we found faster tumor formation kinetics with analogous histological features in secondary recipients (Table 1).

We could not evidence effective metastasis formation in either subcutaneous or orthotopic implantation assays. Moreover, we injected 3H-Fos cells into tail vein of NOD/SCID mice and could not detect tumor formation in any mouse tissue, neither in bioluminescence assays nor in immunohistochemistry studies (Fig. 5i–k). Therefore, we recovered GFP-positive cells from bone marrow of 3H-Fos tumor-bearing mice and injected them into tail vein of secondary recipient mice (Supplementary Fig. S5c–e). This approach yields tumor formation in one out of five mice and in different tissues observed by bioluminescence, FACS analysis and immunofluorescence techniques.

These data indicate that c-Fos expression promotes *in vivo* tumorigenesis in immortalized hMPCs, and that inoculation generates chondrogenic tumors in both heterotopic and orthotopic location.

### c-Fos expression in transformed murine MPCs generates chondrogenic tumors

**Figure 6:**
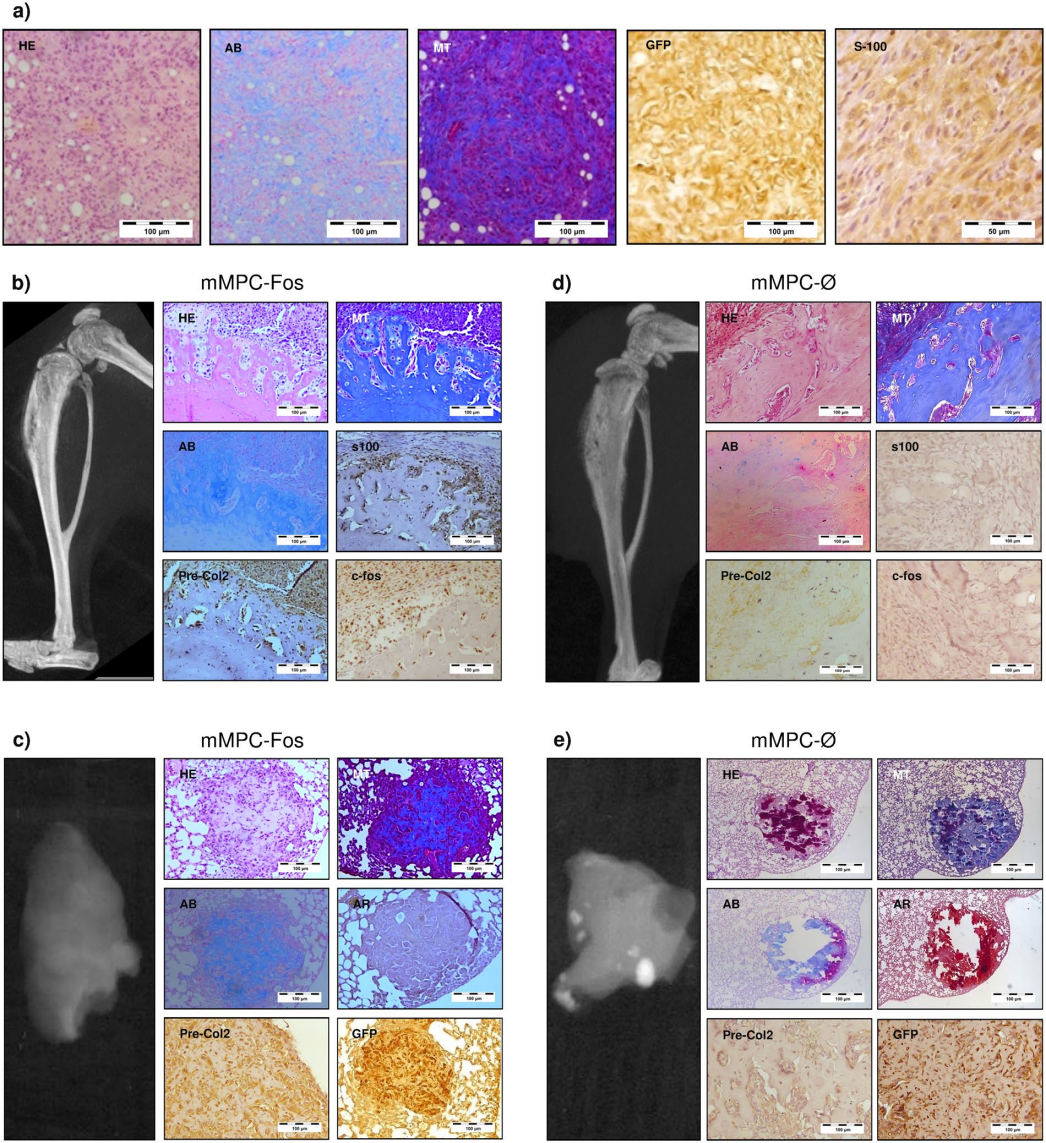
c-Fos expression in murine p53−/−Rb−/− MPCs generate chondrogenic tumors. (**a**) Characterization of tumors generated after subcutaneous implantation of mMPC-Fos cells (n = 3). (**b**) Characterization of tumors generated after intratibial implantation of mMPC-Fos cells (n = 6). (**c**) Non-calcified lung metastasis generated by mMPC-Fos tumors in intratibial implantation model (n = 5). (**d**) Characterization of tibia samples implanted with mMPC-Ø cells (n = 3). (**e**) Calcified lung metastasis generated by mMPC-Ø tumors in intratibial implantation model (n = 3). Alcian blue (AB) and Masson’s trichrome (MT) indicates acid mucin and typical chondrogenic matrix deposition. Chondrogenic phenotype generated by mMPC-Fos was also confirmed by IHC of specific markers expression (Pre-Col2, S100). Note that lung metastasis from mMPC-Fos also appears typically chondrogenic and no calcium deposition is detected as shown by loss of staining for Alizarin red (AR), while tumors generated by mMPC-Ø appears as an ossifying tumour showing direct bone formation and also metastatic disease shows radio dense and osteogenic characteristics.

Once the effect of c-Fos in human MPC transformation was assessed, we moved to corroborate our findings in terms of chondral tumor phenotype related to c-Fos expression. A previous paper shows tumors arising from transformed mouse p53KO mMPCs switch their phenotype from osteoblastic to chondroblastic when c-Fos is expressed in them^26^. We have previously reported that immortalized p53−/−Rb−/− mMPCs originate OS when inoculated orthotopically with the ability to form calcified metastatic nodules in lung tissue^9^. These double mutant transformed murine cells have mutations functionally equivalent to those present in our 3H immortalized hMPCs model. We thus wondered whether c-Fos could promote a switch in tumor phenotype and develop chondrogenic tumors also in already transformed p53−/−Rb−/− mMPCs murine cells. After p53−/−Rb−/− mMPCs transduction with c-Fos lentiviral vector (mMPC-Fos), the transgene expression was confirmed (Supplementary Fig. S6) and mMPC-Fos were implanted both subcutaneously and orthotopically as described above (Fig. 6 and Table 1). Histology of subcutaneous tumors shows the expression of chondrogenic markers (Fig. 6a). Moving to orthotopic model, x-ray studies revealed osteolytic lesions in mice inoculated with mMPC-Fos (Fig. 6b) and lung metastasis in these mice, which showed the expression of chondroblastic markers and no calcification, as assessed by x-ray and Alizarin Red staining (Fig. 6c). In contraposition, the tibias injected with control p53−/−Rb−/− mMPCs (mMPC-Ø) showed the expected osteogenic tumors (Fig. 6d) and calcified metastasis (Fig. 6e).

In sum, these results indicate that, similar to immortalized human MPCs, c-Fos expression in transformed murine MPCs promotes the formation of tumors with chondrogenic phenotype.

## Discussion

c-Fos is a known oncogene expressed in sarcoma tumors, whose deregulated expression is enough to transform a number of cell types, including cells of mesenchymal origin as fibroblasts^31^. In this report, we investigate the hypothesis of MPCs as possible cells of origin in sarcoma context, using c-Fos expression as cell-transforming hit.

Transgenic mice overexpressing c-Fos from ubiquitous promoters develop OS postnatally, suggesting selective targeting of osteogenic precursors by this factor^5,32^. Conversely, tumors originated in c-Fos chimeric mice show clear chondrogenic phenotype, indicating preferential c-Fos targeting to chondrogenic cells^25^. These discrepancies have been explained by differential transgene expression kinetics in each case. Hence, in transgenic mice exogenous c-Fos is detected postnatally, whereas in the case of chimeras, transgene expression starts at E13.5^5,25^. It has been proposed that c-Fos could target particular subsets of cells at sequential differentiation stages to give rise to different tumor types^25^. However, this could also reflect transformation of a shared precursor for both OS and CS, where c-Fos would lead to the development of different transcriptional programs at specific differentiation steps^33^. In this regard, a common embryonic progenitor has recently been described for osteoblasts and a subset of adult MPCs^34^. Forced expression of c-Fos in mouse embryo limb bud at E10 causes chondrodysplasia^35^, thus indicating a common target cell type in this model and in c-Fos chimeras.

In human bone tumors, c-Fos expression has been already reported in OS tumor^36^, but less extensive is its study in chondrogenic tumors. A previous report claims that c-Fos expression is restricted to bone-forming lesions, while cartilaginous tumors is devoid of immunoreactivity^27^. By contrast, other authors find that most chondrosarcomas show moderate to extensive levels of c-Fos expression^28,37,38^. Here, using similar methodology than mentioned studies, we show c-Fos expression by immunohistochemistry in both, OS (49%) and CS (76%). Moreover, we provide evidence that c-Fos is expressed at RNA level in different benign and malignant cartilage tumors (Fig. 1). Interestingly, we have been able to define genes related to c-Fos expression pattern and therefore define a number of cell processes related to c-Fos in human sarcoma context, suggesting a possible clinical relevance of c-Fos expression in these tumors.

Whereas transgenic OS originate from areas close to long bones periosteum^25^, most chondrogenic tumors develop adjacent to joints and in the intramedullary region^25,33^. MPCs reside in bone marrow and have the potential to differentiate into osteoblastic, adipocytic and chondroblastic lineages. These cells have been proposed as cells of origin for a number of sarcomas^3,9,13,30,39,40^; however, their possible role in c-Fos-induced CS, OS, or both had not been addressed so far, especially in a human context. Therefore, in order to define any possible role of mesenchymal progenitor cells as the cell of origin of sarcoma tumors in human context, we induced the expression of c-Fos in human MPCs and we tested cell behavior, transformation and tumorigenic ability. Human MPCs require several oncogenic events to acquire transformed phenotype^12^; accordingly, expression of c-Fos alone did not achieve oncogenic transformation of primary hMPCs. We then employed immortalized hMPCs, which accumulate oncogenic modifications in hTERT and HPV-16 E6/E7 genes but still do not display tumorigenic features^12^. These cells were effectively transformed *in vitro* and acquired tumorigenic capacity *in vivo* upon induction of c-Fos expression, thus confirming c-Fos transforming potential in hMPCs. Interestingly, data show the transformation mechanism is not related to c-MYC and H-RAS, which were the pathways altered in a previous report^12^. Moreover, *in vitro* and *in vivo* data, both in human and mouse MPC context, suggest that 3H-Fos cells retain chondrogenic differentiation ability, thereby yielding tumors with chondrogenic phenotype.

Our data indicate that c-Fos induces higher proliferation rate of both primary and immortalized hMPCs; this correlates with an increase in the percentage of cells in S and M phases, and an increment in B-Myb and cyclin A expression in 3H-Fos comparing with 3H-Ø. C-Fos induces proliferation also in other cell types by modulating expression levels of cyclin D1 and cyclin A, which are cell cycle regulators with CRE and AP-1 consensus binding sequence in their promoters^41^. Hence, c-Fos promotes chondrocyte growth through transcriptional activation of cyclin D1^41^. Likewise, high levels of cyclin D1 are detected in c-Fos-induced OS^42^. In contrast murine osteoblast cell lines undergo S-phase entry and increased proliferation upon c-Fos overexpression, with a parallel induction of cyclins A and E, but with no modification of cyclin D levels^43^.

We observed *In vitro* cell transformation due to c-Fos expression in immortalized hMPCs. Cell-transformation process is linked to impaired cell-cycle regulation, as discussed before, and also to other cellular processes as, for example, increased cell survival and a bioenergetics switch^44^. Specifically, mitochondrial dysfunction has been reported as important cellular process for other oncogene-overexpression induced cell transformation models. Here we observed mitochondrial network rearrangement and impaired oxidative phosphorylation related to mitochondrial dysfunction in 3H-Fos. Moreover, some OS cell lines, as LM7 and 143B, also present similar mitochondrial imbalance and metabolic features^45^.

Also at intracellular level, we found profound changes in cytoskeleton structure after c-Fos expression. It is well known actin cytoskeleton is related to cell shape and mechanical properties of the cell, including cell motility processes^46^. When we tested cell function, we observed reduced mobility due to c-Fos expression and we concluded 3H-Fos cells are unable to properly generate actin polymerization and microtube formation, thereby reducing their ability to move and migrate. In this sense, a previous report indicates that endogenous c-Fos downregulation results in an increase in migration and invasion capacities of trophoblast cells, suggesting an inhibitory role for c-Fos in these processes^47^.

Our results show that c-Fos expression in MPCs from human origin causes an imbalance in differentiation potential of these cells, favoring chondrogenic fate. c-Fos is largely but transiently overexpressed in the initial steps of osteogenic differentiation, while it is downregulated in late osteogenic differentiation stages^48^. Conversely, c-fos has no apparent role during initial phases of chondrogenesis^49^, while in late stages is expressed and controls chondrocyte metabolism. Indeed, c-Fos *in vivo* is constitutively expressed in chondrocyte nuclei^50^, while *in vitro* it has been shown c-Fos is a critical downstream effector of IL-1, FGF, IGF and PTHrP, thereby suggesting c-Fos has a pivotal role in chondrocyte proliferation and differentiation regulating pathways^51,52^. A recent study suggests c-Fos in p53KO mouse MPCs induces the expression of SOX9, a master regulator of chondrogenesis, and downregulation of RUNX2, a master regulator of osteogenesis^26^. In our human cell model, we observe the same overexpression of SOX9 and downregulation of RUNX2 in 3H-Fos cells. Altogether, data suggest a constitutive expression of c-Fos in MSCs impedes the progression into osteogenic differentiation, while promotes initial stages of chondrogenesis via modulation of SOX9 expression. Thereafter, c-Fos itself may be related in an induction of proliferation of chondrocytes in their late stages of differentiation.

Our data indicate that expression of c-Fos in immortalized hMPCs promotes the development of chondrogenic tumors when these cells are inoculated in mice. Although 3H-Fos derived tumors are predominantly chondrogenic, sporadic areas of osteoid were observed in histological preparations. This fits with the phenotype of chondrogenic tumors derived from c-Fos chimeric mice, where sparse areas of osteoid coexist with prevalent expression of chondrogenic markers^25^. We assessed the c-Fos effect in tumor phenotype in transformed mMPCs, and the procedure yields tumors with chondral phenotype strongly resembling the phenotype found elsewhere in tumors raised from other mouse transformed cells expressing c-Fos^26^.

We were not able to detect metastasis in any primary mice implanted with human transformed 3H-Fos cells. Moreover, we observed no engraftment in distant sites when 3H-Fos cells were implanted in bone orthotopic model, even do we observed 3H-Fos cells in lung tissue after injection, suggesting a systemic spreading of cells due to injection model. It is well-established that secondary recipient mice generate faster and more aggressive tumors, a process linked to clonal selection during tumor formation and cancer progression^53^. Following that idea, here we performed serial xenotransplants using 3H-Fos cells injected in orthotopic and subcutaneous models and obtained lower tumor-forming latencies in secondary mice. That encourages us to study possible metastatic events in secondary recipient mice. Notably, we observed metastatic tumor formation only in one animal and long term after iv injection of orthotopic tumor-derived 3H-Fos cells, suggesting once again poor metastatic ability of 3H-Fos cells. In this sense, Fittall *et al*.^54^ reported recurrent rearrangement of FOS only in osteoblastoma benign tumors and not in other malignant bone tumors, concluding that in clinic FOS may be genetically altered only in benign bone tumors.

It is worth note here cell models used in this study have multiple limitations. The specific combination of oncogenic events used in human cells may not resemble a clinical scenario, while some of the data are related to a mouse cell model.

In this article, we provide evidence for the role of MPCs as cells of origin of c-Fos-induced CS. Clinical significance of our findings is emphasized by the presence of c-Fos expression in vast majority of human CS samples analyzed. Our findings may contribute to the identification of early events in sarcoma development, which set the stage for novel therapeutic approaches.

## Materials and Methods

### Sarcoma datasets and comparative expression analysis in tumor mRNA samples

Comparative expression analysis was performed using R2: Genomics Analysis and Visualization Platform (http://r2.amc.nl). Sarcoma datasets were selected and specific gene expression data were extracted from them. In supplementary information file, there is specific information related to used datasets.

### Cell culture

Primary bone marrow hMPCs were purchased from Lonza. Immortalized hMPCs were kindly provided by Dr. Funes. p53−/−Rb−/− mMPCs were obtained as previously described^9^. All cells were cultured in DMEM supplemented with 10% FBS, 1% penicillin/streptomycin and 2mM L-glutamine. Human cells (3H, 3H-Ø and 3H-Fos) cells were validated by Short Tandem Repeat (STR) profiling using the PowerPlex 16HS system and cells were all found to show the same unique profile.

### Lentiviral production and cell transduction

c-Fos cDNA was purchased from imaGenes (clone IRAUp969F0824D,), related to the NCBI c-fos sequence BC004490.2. c-fos cDNA was excised, cloned and sequenced into the pWPI (Addgene plasmid, 12254) and pOTB7 plasmids, which carry Tomato and GFP genes respectively as reporter fluorescent proteins. Lentiviral particles were produced by transient calcium phosphate transfection of HEK-293 cells. 48 hours after transfection, supernatants were collected, concentrated by ultracentrifugation, and stored at −80 °C. hMPCs were transduced with pOTB7 plasmid-derived lentiviral particles, while mMPCs were transduced with pWPI plasmid-derived lentiviral particles. Tomato and GFP expression were assessed by flow cytometry. Expression were also confirmed via qRT-PCR, western blot and immunofluorescence. Cells transduced with lentivirus coding only for fluorescent proteins were used as controls. Expression data are available in the Gene Expression Omnibus (GEO) data repository under the accession number GSE79158.

### Animal procedures

Surgical procedures and animal care were performed with the approval of the Animal Research and Welfare Ethics Committee (Comité de Ética de la Investigación y de Bienestar Animal) of Instituto de Salud Carlos III, following EU and Spain Directives for animal experiments and in a specific pathogen-free environment. 10 to 15 week-old immunodeficient NOD.CB17-Prkdcscid (NOD-SCID) mice were inoculated either subcutaneously (10^6^ cells/mouse), intravenously (5 × 10^6^ cells/mouse) or intra-bone marrow (5 × 10^5^ cells/mouse). Prior to inoculation, cells were resuspended in PBS and filtered through a 70 µm nylon filter. For intra-bone marrow administration, mice were anesthetized with 2% isofluorane. Alternatively, anesthesia was induced by intraperitoneal injection of a mixture of xylacine (Rompun, Bayer)-ketamine (Imalgene 1,000, Merial). The right hind limb was bent 90  to allow drilling tibial plateau with a 25G needle before cell inoculation with a 27G needle.

### Statistics

Every experiment is shown at least in triplicate and is representative of three independent experiments. In all cases, the n is provided in the figure legend. Data were expressed as means ± standard deviation (SD). T-Student and ANOVA analysis were performed using Graph Pad Prism 6 software. P-value is indicated in each panel and figure (*p ≤ 0.05; **p ≤ 0.01; ***p ≤ 0.001).

### Statement of Significance

The study of tumor-initiating cells is of great interest based in the hypothesis that they would be responsible for tumor onset. We demonstrate here that expression of c-Fos, an oncogene present in most biopsies from patient-derived chondrosarcomas, induces transformation in immortalized mesenchymal progenitor cells (human and murine) generating tumors with chondrogenic phenotype. Therefore, data indicate a main role of c-Fos in chondrosarcomas originated by mesenchymal progenitor cells.

### Disclaimer

The content is solely the responsibility of the authors and does not necessarily represent the official views of the ISCIII.

## Electronic supplementary material

Supplementary Information

Supplementary Figure S4
