# Supplementary material for: c-Fos induces chondrogenic tumor formation in immortalized human mesenchymal progenitor cells
Source: Sci Rep. 2018 Oct 23;8:15615. doi: 10.1038/s41598-018-33689-0 (PMC6199246; doi:10.1038/s41598-018-33689-0)
Supplement: Supplementary file 1 — Supplementary Information [file 41598_2018_33689_MOESM1_ESM.pdf]

c-Fos induces chondrogenic tumor formation in immortalized human mesenchymal progenitor cells.

Ander Abarrategi[1,2*], Stefano Gambera[1*], Arantzazu Alfranca[1], Miguel A. Rodriguez-Milla[1], Raquel Perez-Tavarez[3], Kevin Rouault-Pierre[2], Alexander Waclawiczek[2], Probir Chakravarty[4], Francisca Mulero[5], César Trigueros[6], Samuel Navarro[7], Dominique Bonnet[2], Javier García-Castro[1].

[1] Unidad de Biotecnología Celular, Instituto de Salud Carlos III, Madrid E-28021, Spain.
[2] Haematopoietic Stem Cell Laboratory, The Francis Crick Institute, London WC2A 3LY, UK.
[3] Unidad de Histología, Instituto de Salud Carlos III, Madrid E-28021, Spain
[4] Bioinformatics Core, The Francis Crick Institute, London, United Kingdom
[5] Molecular Image Core Unit, Spanish National Cancer Research Centre. Madrid E-28029, Spain.
[6] Mesenchymal and Hematopoietic Stem Cell Laboratory, Fundación Inbiomed, San Sebastian, E-20009, Spain.
[7] Pathology Department, University of Valencia, Valencia E-46010, Spain.

* Equal contribution.

SUPPLEMENTARY FIGURES LEGENDS

Supplementary Figure S1. a-b, Induction of c-Fos expression in hMPCs. **a**, proliferation rate of primary hMPCs after transduction with c-Fos or with empty vectors (n=3). **b**, β-gal positive staining of primary hMPCs after mid-term in vitro culture (n=3). **c-e,** Induction of c-Fos expression in 3H cells. **c,** FACS histograms. White area indicates isotype control and grey area represent specific antibody labelled sample. First row shows data from 3 different primary hMPCs, the second row shows data from 3H-Ø cells and the third-row show expression in 3H-Fos cells (n=3). **d**, examples of FACS plots of cell cycle studies shown in the main figure 2D (n=3). **e,** examples of FACS histograms of cyclin A1 expression (n=3).

Supplementary Figure S2. **a-f**, Gene expression studies. Raw data of the triplicate study is accessible in GEO data repository (http://www.ncbi.nlm.nih.gov/geo/) under the accession number GSE79158. **a**, Representation of genes with differential gene expression. **b-c**, functional enrichment analysis. **b,** Examples of the gene ontology terms obtained analyzing the upregulated and downregulated genes separately (WebGestalt GSAT). **c,** Examples of the gene ontology terms obtained analyzing whole dataset (GSEA). **d,** RT-qPCR assays in specific genes performed to validate the RNA microarray results (n=3 in each case). **e,** Example of an enriched GO term. **f,** Other GO terms related to cell survival, apoptosis and senescence. G-H, Thermal stress functional study. **g,** examples of the apoptosis observed in the thermal stress study performed. **h,** Quantification of apoptotic cells after the heat shock (n=3). (unpaired t-test. *$p \leq 0.05$; **$p \leq 0.01$; ***$p \leq 0.001$).

Supplementary Figure S3. Gene expression study related to H-RAS and c-myc signaling pathways in 3H-Fos cells. Data show differences in RNA expression (3H-Fos cells vs 3H- Ø cells) (n=3) expressed as fold change (Log2). Green line indicates significant downregulation (limit log2,X ≤ -2) and red line indicates significant upregulation (limit

log2,X ≥ 2). Note none of the genes shown in this figure reach the threshold of significant up or downregulation limit. A) Chart showing data for RAS family genes and MYC family genes and PP2A subunits. Main genes of interest are highlighted in bold. B-C) Group of genes obtained from Gene Ontology terms (GO terms) related to H-RAS and c-myc pathways. None of the GO terms was statistically significant. B) Chart summarizing the list of genes grouped in the GO terms "Biocarta RAS pathway", "HRAS targets" and "HRAS signaling via NFKB". C) Chart summarizing the list of genes grouped in the GO term "Hallmark MYC targets".

Supplementary Figure S4. Representative videos of 3H-Ø cells (left) and 3H-Fos cells (right).

Supplementary Figure S5. a-b, Bone phenotype in 3H-Fos cells induced tumors. **a**, histological detail and bone associated markers in tumors generated by subcutaneous inoculation of 3H-Fos cells. **b**, Similar study in tumor arise from intratibially injected 3H-Fos cells. (HE, Hematoxilin/Eosin: OC, Osteocalcin: ON, osteonectin). **c-e,** 3H-Fos cells i.v. implanted after primary bone-tumor formation (N=5). **c**, schematic represents the cell harvesting from tibia tumors and further i.v. implantation protocol. **d,** bioluminiscence images of whole mouse at three different time points showing metastatic events at end point. **e,** bioluminiscence images of harvested tissues from the metastatic mouse show luminescent areas in lung, forelimb and muscle tissue in legs. FACS study shows the presence of GFP positive cells in Bone marrow tissue. IHC confirms the presence of implanted human cells in these tissues (hV, Vimentin).

Supplementary Figure S6. a-b, Assessment of cells transduction and c-Fos expression in mMPCs. **a**, Representative cell cytometry histogram showing GFP expression in 3H parental (light grey, no GFP expressed) and c-Fos transduced cells (dark grey). **b**, Representative western blot of c-Fos showing parental cells (mMSCp53-/-Rb-/-), cells

transduced with the empty vector (mMSCp53-/-Rb-/-Ø), and cells transduced with c-Fos expression vector ((mMSCp53-/-Rb-/-FOS) (n=3).

Suppl. Figure 1

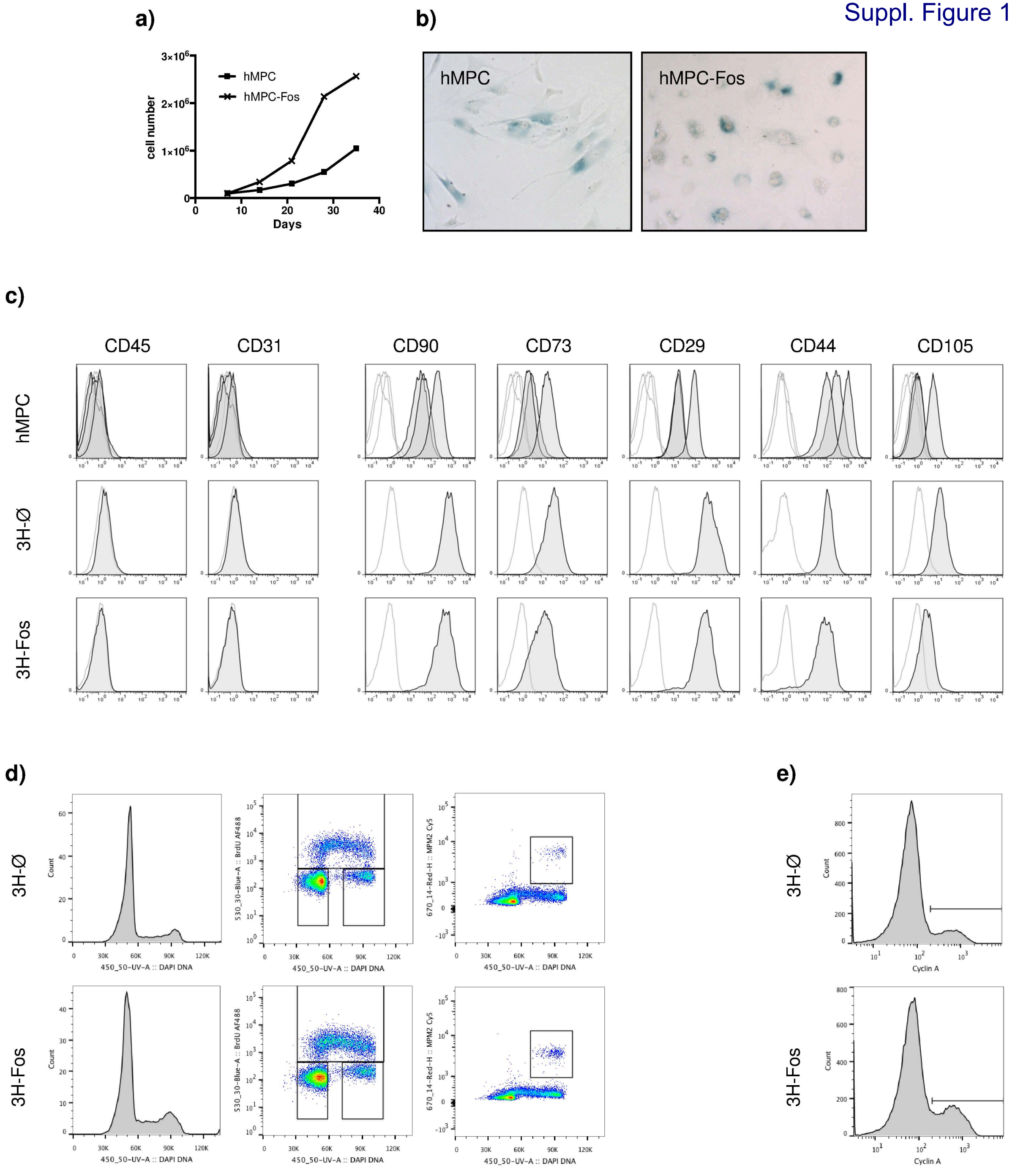

Suppl. Figure 2

a)

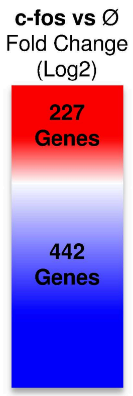

b)

Terms from Upregulated genes (227):

P value	N genes	GO Term
5.49E-02	25	Cell proliferation
5.49E-02	70	Cell communication

Terms from Downregulated genes (442):

P value	N genes	GO Term
6.47E-27	86	Extracellular region
3.96E-16	115	Receptor signaling pathway
5.55E-05	17	Cytokine activity

c)

GO terms positively correlated to 3HF cells:

FDR p-value	NES	N genes	GO Term
1.7E-04	2.15	67	Meiotic Recombination
3.6E-04	2.101	70	RNA_poll_transcription

GO terms negatively correlated to 3HF cells:

FDR q-value	NES	N genes	GO Term
0	0.278	96	Extracelular matrix
2.7E-04	0.597	99	Metasis
2.9E-02	0.466	178	Cell migration

d)

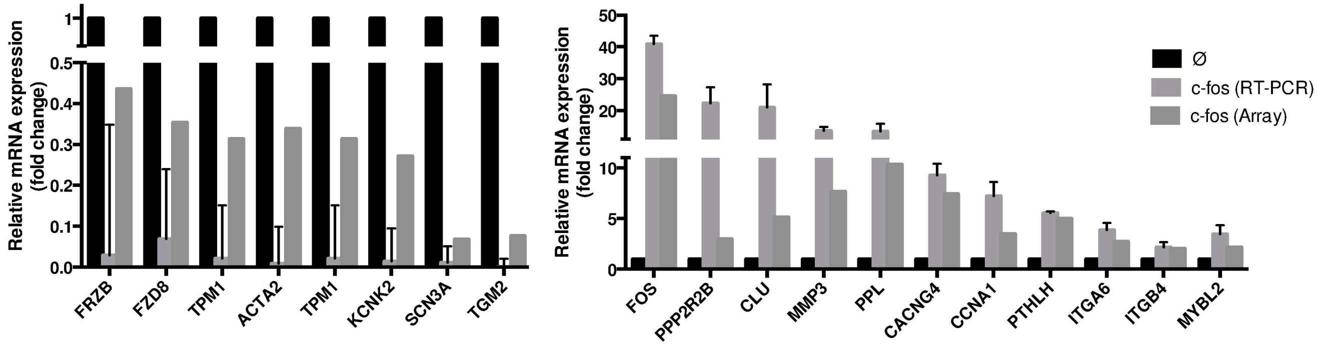

e)

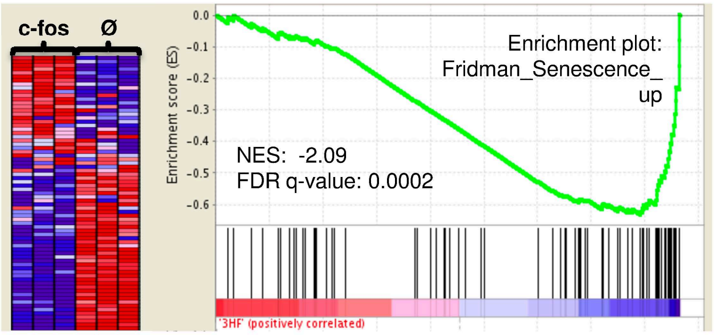

f)

GO terms Negatively correlated to 3HF cells:

FDR p-value	NES	N genes	GO Term
2E-04	-2.09	73	Fridman senescence up
4.9E-03	-1.89	519	Apoptosis by serum deprivation
5.9E-03	-1.88	538	RB1 Targets senescence

g)

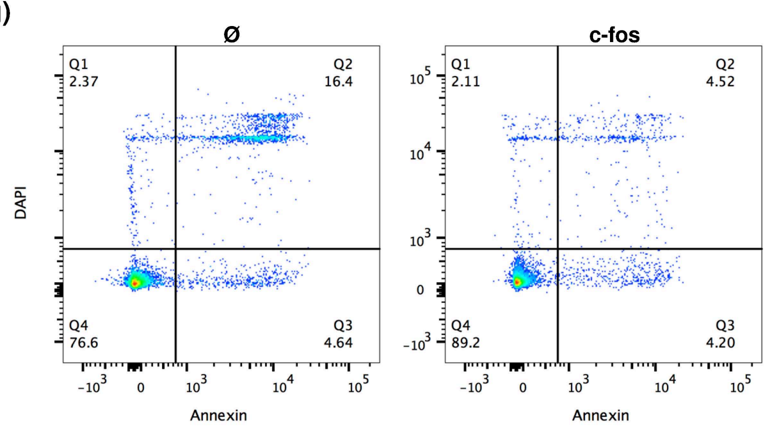

h)

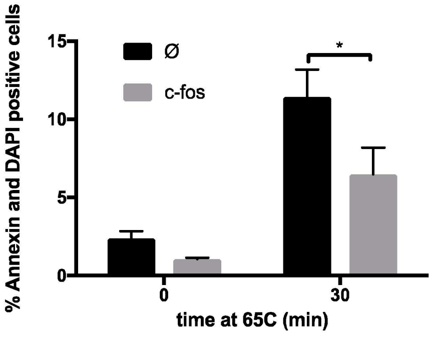

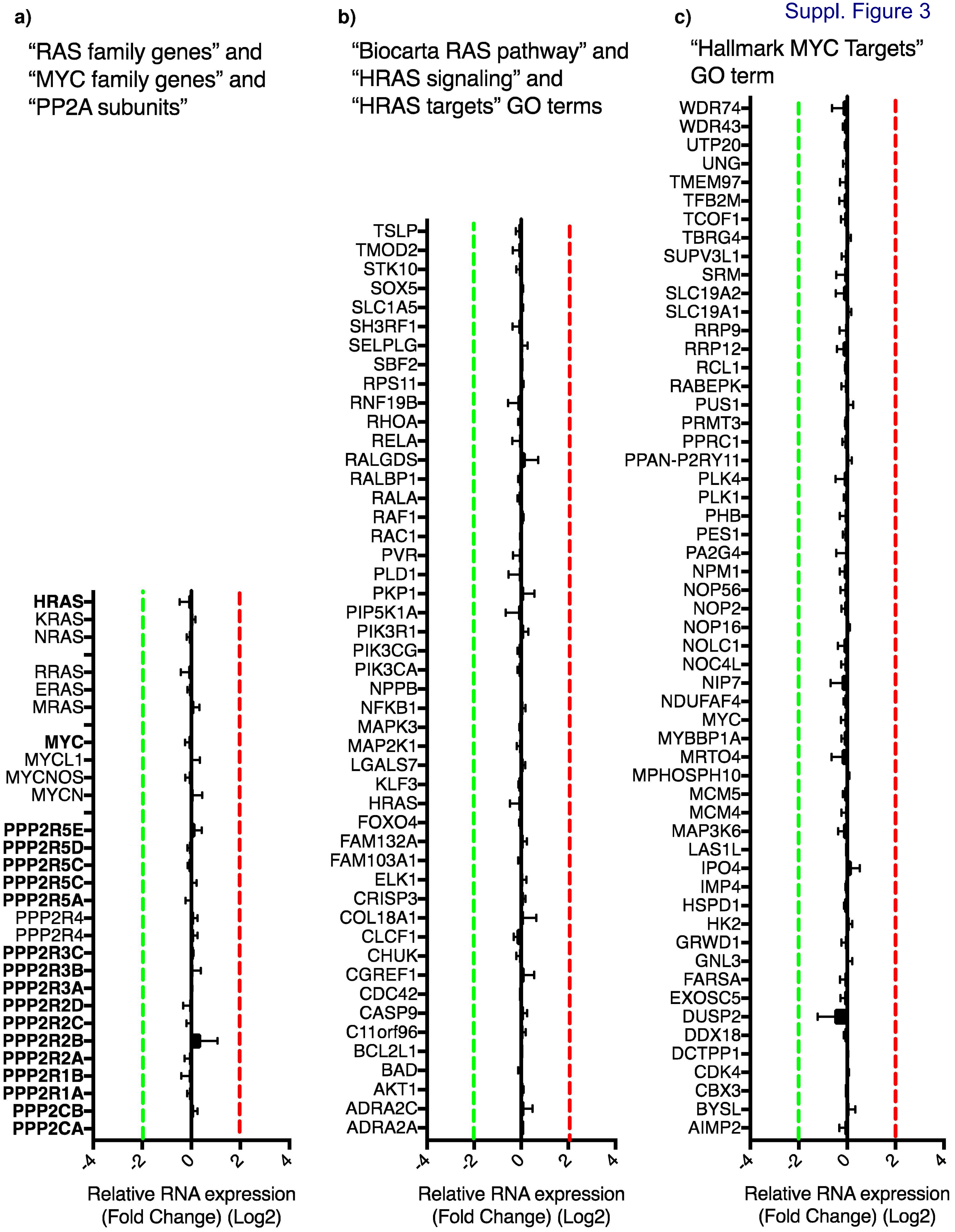
a)
"RAS family genes" and
"MYC family genes" and
"PP2A subunits"
HRAS
KRAS
NRAS
RRAS
ERAS
MRAS
MYC
MYCL1
MYCNOS
MYCN
PPP2R5E
PPP2R5D
PPP2R5C
PPP2R5C
PPP2R5A
PPP2R4
PPP2R4
PPP2R3C
PPP2R3B
PPP2R3A
PPP2R2D
PPP2R2C
PPP2R2B
PPP2R2A
PPP2R1B
PPP2R1A
PPP2CB
PPP2CA
-4
-2
0
2
4
Relative RNA expression
(Fold Change) (Log2)
b)
"Biocarta RAS pathway" and
"HRAS signaling" and
"HRAS targets" GO terms
TSLP
TMOD2
STK10
SOX5
SLC1A5
SH3RF1
SELPLG
SBF2
RPS11
RNF19B
RHOA
RELA
RALGDS
RALBP1
RALA
RAF1
RAC1
PVR
PLD1
PKP1
PIP5K1A
PIK3R1
PIK3CG
PIK3CA
NPPB
NFKB1
MAPK3
MAP2K1
LGALS7
KLF3
HRAS
FOXO4
FAM132A
FAM103A1
ELK1
CRISP3
COL18A1
CLCF1
CHUK
CGREF1
CDC42
CASP9
C11orf96
BCL2L1
BAD
AKT1
ADRA2C
ADRA2A
-4
-2
0
2
4
Relative RNA expression
(Fold Change) (Log2)
c)
"Hallmark MYC Targets"
GO term
WDR74
WDR43
UTP20
UNG
TMEM97
TFB2M
TCOF1
TBRG4
SUPV3L1
SRM
SLC19A2
SLC19A1
RRP9
RRP12
RCL1
RABEPK
PUS1
PRMT3
PPRC1
PPAN-P2RY11
PLK4
PLK1
PHB
PES1
PA2G4
NPM1
NOP56
NOP2
NOP16
NOLC1
NOC4L
NIP7
NDUFAF4
MYC
MYBBP1A
MRTO4
MPHOSPH10
MCM5
MCM4
MAP3K6
LAS1L
IPO4
IMP4
HSPD1
HK2
GRWD1
GNL3
FARSA
EXOSC5
DUSP2
DDX18
DCTPP1
CDK4
CBX3
BYSL
AIMP2
-4
-2
0
2
4
Relative RNA expression
(Fold Change) (Log2)

Suppl. Figure 5

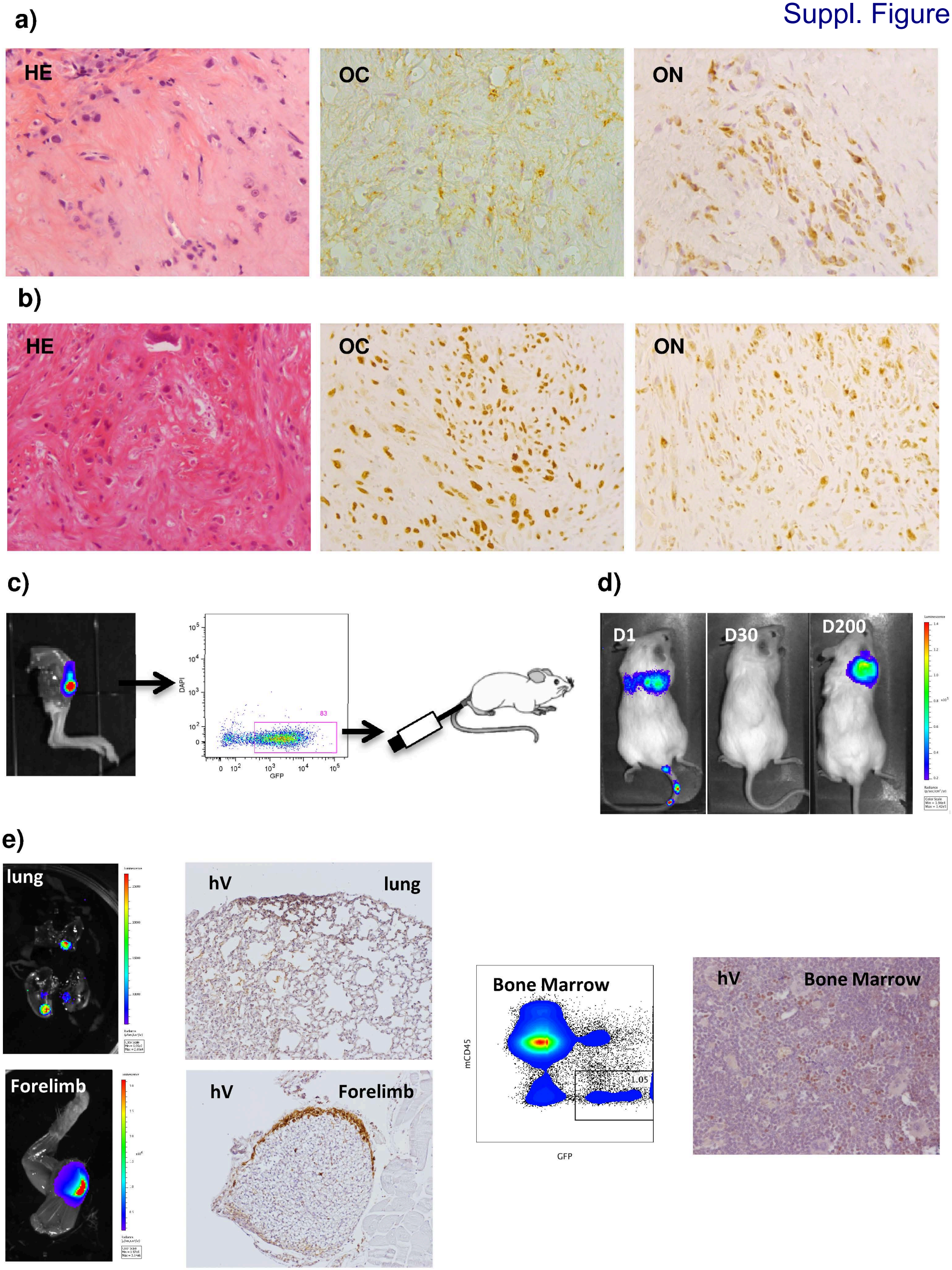

Suppl. Figure 6

a)

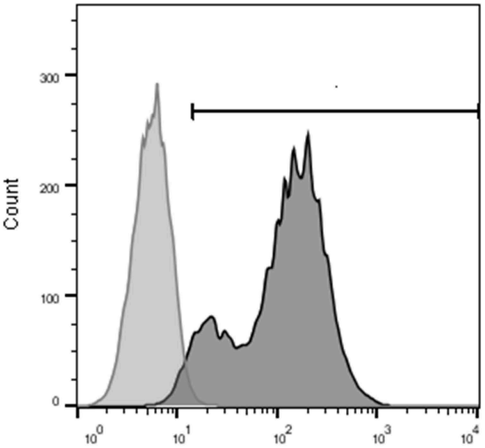

b)

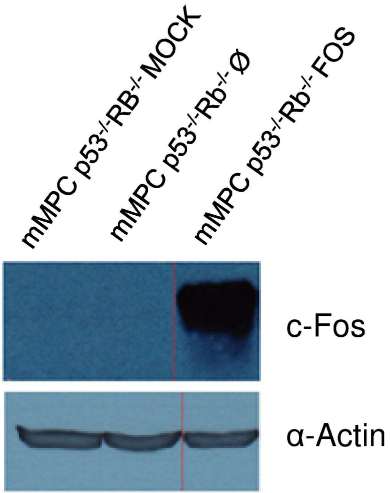

SUPPLEMENTARY MATERIALS AND METHODS.

Sarcoma datasets and comparative expression analysis in tumor mRNA samples:

Dataset 1 corresponds to "Tumor sarcoma-TCGA" dataset (http://cancergenome.nih.gov/). It is a compilation of 259 sarcoma samples. Dataset 2 corresponds to GSE30844 dataset available at Gene Expression Omnibus (GEO). It is a compilation of 21 enchondroma and chondrosarcoma tumors with 2 control growth plate samples, and accessible in the following link, (http://www.ncbi.nlm.nih.gov/geo/geo2r/?acc=GSE30844&platform=GPL6884).

Dataset 3 correspond to "Tumor Sarcoma Mesenchymal – Boshoff" dataset (http://www.ebi.ac.uk/arrayexpress/experiments/E-MEXP-353/). It is a compilation of 96 sarcoma samples. Dataset 4 corresponds to GSE42352 dataset available at Gene Expression Omnibus (GEO). It is a compilation of 127 osteosarcoma samples with survival information, and accessible in the following link,

https://www.ncbi.nlm.nih.gov/geo/query/acc.cgi?acc=gse42352.

All datasets were analyzed using online R2: Genomics Analysis and Visualization Platform (http://r2.amc.nl).

Immunostaining:

Human tissue micro-arrays (TMAs) were prepared from patient's samples in the Pathology Department of University of Valencia. Briefly, TMAs were constructed using the manual Beecher instrument (Silver Springs MD). Two cylinders of each case were assembled. Additional osteosarcoma and chondrosarcoma TMAs were purchased from US Biomax Inc. and tested in this study. Specific information related to TMAs and patients included in the studies could be provided under request or in the US Biomax Inc. website (reference OS803 and T261).

Immunohistochemistry (IHC) and immunofluorescence (IF) studies were performed by standard methods; heat antigen retrieval was performed when required in IHC studies. See primary unconjugated antibodies employed in the table attached at the end of this document. Secondary fluorescent antibodies were from Invitrogen and secondary biotinylated antibodies were from Jackson. Texas Red-X phalloidin (Invitrogen) was used for actin staining in IF studies.

Western blot analysis:

Proteins were extracted with lysis buffer (1% NP-40, 50 mM Tris pH 7.5, 10mM tetrasodium pyrophosphate, 150 mM NaCl, 10 mM NaF, 1 mM PMSF, 5 mM NaF, 20 mM β-glycerophosphate, 0.1 mM sodium orthovanadate and 1:100 protease inhibitor cocktail from Sigma) and quantified using the DC Protein Assay system (Bio-Rad). Proteins (50 μg per lane) were separated in a 10% SDS–PAGE gel and blotted onto PVDF membranes (Bio-Rad). Blots were incubated with primary and secondary antibodies and detected using Immobilon Western Chemiluminescent HRP Substrate (Millipore).

Real-time quantitative RT-qPCR:

First strand cDNA synthesis was performed using 2 μg total RNA with 0.5 μg oligo(dT) (Invitrogen) and 200 U M-MLV reverse transcriptase (Invitrogen). Amplification was done using 10 pmol of each of forward and reverse primer (see the table attached at the end of this document) and the LightCycler 480 SYBR Green I Master mix (Roche), following manufacturer's instructions. Reaction was performed and analyzed on the LightCycler 1.5 480 Real-Time PCR System (Roche). Data was analyzed using the second derivative maximum method to identify the crossing point (Cp) in the LightCycler software 3 (Roche Diagnostics). Housekeeping gene GAPDH was employed as internal control for normalization.

Flow cytometry studies:

Cells (10^5 per labeling) were treated with FcR blocking reagent (Miltenyi Biotec) during 15 minutes and incubated at 4°C in the dark for 30 min with each antibody or its respective isotype control. Then, stained cells were washed in PBS. When needed, cells were subsequently incubated with a fluorochrome-conjugated secondary antibody at 4°C in the dark during 30 min and washed in PBS. Finally, cells were analyzed in a FACSCalibur flow cytometer (BD) and data were visualized with FlowJo 7.6.5 program.

Cell senescence, cell proliferation, and cell cycle assays:

Senescence-associated β-galactosidase staining was performed with Senescence Cells Histochemical Staining kit (Sigma), following the manufacturer's protocol. For proliferation assessment, $5x10^4$ cells were seeded in 25 cm^2 flasks. Cells were tripsinized, every 4 days, counted, and replated in the same conditions. Then duplication rate and doubling time were calculated. In cell cycle experiments, 60% confluence cells were treated with BRDU during an hour, fixed (Ethanol 70%), permeabilized (triton 0.2%) and processed for detection with BRDU or MPM-2 antibodies; DAPI was used for DNA labeling. 60% confluence cells were fixed (PFA 2%), permeabilized (triton 0.2%), and processed for detection with Cyclin A1 antibody. All cells were analyzed in a BD LRSII flow cytometer.

Global transcriptional analysis and study:

RNA was extracted from cells 10 days after transfection. 250 ng of total RNA from each biological sample were processed and the transcriptional profile was analyzed with the use of the GeneChip Affymetrix platform and the GeneChip Human Gene 2.0 ST Array. Specific protocols, details and expression data are available in the Gene Expression Omnibus (GEO) functional genomics data repository under the accession number GSE79158. Data were analyzed using Bioconductor 2.12 (http://www.bioconductor.org) running on R 3.0.0. Probeset expression measures were calculated with the Affymetrix package's Robust Multichip Average (RMA) default

method. Enrichment of pathways and biological gene sets, downloaded from MSigdb version 5.1 (http://software.broadinstitute.org/gsea/msigdb) were assessed using GSEA. Gene sets with an enrichment q value of less than 0.05 were judged to be statistically significant. Differentially expressed genes were assessed between samples using an empirical Bayes t test (limma package) and p values were adjusted for multiple testing by the Benjamini-Hochberg method (1). Any probe sets that exhibited an adjusted p value of 0.05 were called differentially expressed. Differentially expressed genes were used to look for geneset enrichment from Gene Ontology Biological processes using the Metacore pathway tool (http://thomsonreuters.com/metacore/). The analysis employs a hypergeometric distribution to determine enriched gene sets. All genes on the Affymetrix chip were used as the background for enrichment analyses. The p value was corrected using the Benjamini and Hocberg multiple testing correction method.

Cellular bioenergetics studies:

Mitochondria membrane potential was measured using TMRE dye that is sequestered by active mitochondria. Cells were plated at 5,000 cells/cm^2 in a 12-Well multiple well dish. After 72h, 40 nM TMRE was added directly to cell culture media, incubated for 25 minutes and then cells tripsinized, washed, incubated with DAPI to further identify non-viable cells and analyzed in a BD LRSII flow cytometer at room temperature. For the determination of cellular oxygen consumption rate (OCR) and extracellular acidification rate (ECAR), assays were performed in the XF Extracellular Flux Analyzer (Seahorse Bioscience). Real time OCR was measured in 96 well plates using an XF Assay Kit (Seahorse Bioscience). Cells (2x10^5 cells per well) were seeded 24h before measurements and media was replaced with assay medium (1mM sodium pyruvate and 10mM Glucose, pH 7.4, Seahorse Bioscience) before testing. Following baseline measurements, serial addition of multiple compounds was executed (1.264 μM oligo-mycin A (Sigma), 0.4 μM FCCP (Sigma), and a mix of 1μM rotenone (Sigma) and 1 μM

antimycin A (Sigma)) and real time OCR measurements were performed. 10 replica wells were used for each cell type and measurement condition. After the study, cells were fixed in ethanol 70%, permeabilized with triton 0.2% and stained with DAPI for total cell number counting per well. OCR is reported in pmoles/minute/cell. Lactate measurements were carried out using the lactate scout+ (EKF Diagnostics). Cells were plated at 5,000 cells/cm^2, media was replaced at 24 hours and lactate was measured directly from cell culture media after 48h incubation. For inhibition of the pentose-phosphate pathway, cells were plated at 5,000/cm^2 in a 48-well multiple well dish. After 24h, fresh media containing 6-Aminonicotinamide (Sigma) was replaced and incubated 72h. Cell counts and viability measurements using DAPI were performed on a MACSQuant VYB (Milteny Biotec).

In vitro agar colony formation assay:

Colony formation in agar was assessed with the Cytoselect 96-well cell transformation assay kit (Cell Biolabs), following manufacturer's instructions. After 7 days, agar was diluted and MTT assay was conducted as specified in the manufacturer's protocol. Data provided correspond to absorbance measurement (570nm-650nm).

Heat shock and hyperthermia stress:

Similar to previously described (2), Cells were seeded in 6 well plates and cultured in normal cell culture conditions (37 °C, 5% CO_2, 95% relative humidity). Confluent cells were transferred to an oven preset to 65°C. After 30 minutes, cells were placed back in normal cell culture conditions and maintained there 20 hours prior to analysis.

Cells were trypsinized, washed twice with cold PBS and resuspended in binding buffer (eBiosciences 00-0055-56) at $1x10^6$ cells/mL. then AnnexinV-PE (eBiosciences BMS306PE/100) was added at 1uL per $1x10^5$ cells and incubated 15 minutes at RT in dark. Cell were centrifuged and resuspended in new binding buffer with DAPI (Sigma, S3023) at 1ug/mL and analyzed in a BD LRSII flow cytometer.

In vitro Cell mobility and Migration Assays:

Cell random mobility was monitored in a SP5 confocal laser scanning microscope (Leica) equipped with a heat chamber for cell culture studies. Recording was performed during 17 hours. Cell tracking was performed using the "Chemotaxis and Migration Tool" program (Ibidi, Germany). For wound healing assays, cells were allowed to reach confluence and a pipette tip was used to make a scratch in cell monolayer. Images were obtained at different time points and wound area was measured in each of them. Data are provided as cell-covered wound area (μm^2). For directed cell migration studies, P24 transwell chambers with 8 mm pore filters (BD) were coated with 0.1% gelatin (Sigma) for one hour at 37°C. Cells were trypsinized and $5x10^4$ cells were transferred to each transwell chamber. For induction of cell migration 10% FBS DMEM was placed in lower chamber. After incubation period, migrating cells were fixed with 10% formaldehyde for 10 min, stained with 0.25% crystal violet in 10% formaldehyde for 10 min, rinsed in water, air-dried and photographed. Then crystal violet was eluted with 0.1 M Sodium citrate in 50% Ethanol, pH 4.2 and absorbance was measured in an Infinity M200 plate reader. Data were normalized to total cell number using a regression curve performed using different amounts of cells. Each experiment was performed by duplicate and repeated three times.

Adipogenic, osteogenic and chondrogenic cell differentiation:

Cells were differentiated using Adipogenic, Osteogenic or Chondrogenic MPCs Differentiation BulletKit (Lonza), following manufacturer's instructions. For the assessment of adipogenic differentiation, cells were fixed in 4% paraformaldehyde and stained with Oil red O (Sigma-Aldrich); osteogenic differentiation was evaluated by Alizarin red S staining (VWR), and chondrogenic differentiation was determined by Alcian blue dye.

Whole body Bioluminiscence:

For bioluminescence studies, cells were transduced with lentiviral vectors which carry both Luciferase and GFP genes (TK-Gluc (Addgene) and LV-SFFVp-Luc) (3). Whole animal bioluminescence was recorded using the IVIS Lumina image system (Perkin Elmer). Mice were anesthetized with 2% isofluorane and, after intraperitoneal administration of 100 μL of a D-luciferin solution (12.5 mg/ml in PBS) mice were imaged in dorsal and ventral positions. Data were analyzed with Living Image software (Perkin Elmer).

Micro Computered Tomography (microCT):

Formalin-fixed samples were imaged in a μCT system (eXplore Vista, GE), with an X-ray tube voltage of 50 kV and a current of 200 μA. The scanning angular rotation was 180°, the angular increment 0.40°, and the voxel resolution 50 μm. Data sets were reconstructed and segmented into binary images (8-bit BMP images) for the subsequent image processing and 3D surface reconstructions using MicroView ABA 2.2 software (GE Healthcare).

Histological processing:

Harvested samples were fixed 24 hours in 10% neutral buffered Formalin and decalcified with 4% hydrogen chloride / 4% formic acid in water during 3 days or alternatively with 17% EDTA (Osteosoft, Millipore) during 7 days. Then samples were processed, paraffin embedded and sectioned (5 μm) for histological studies. Hematoxylin/eosin, Masson's trichrome, and Alcian blue stainings were performed. Osteoclast identification was conducted using TRAP staining kit (Sigma) in EDTA decalcified samples, according to manufacturer's protocol. Immunostaining of histology samples was performed has been defined previously. See primary

unconjugated antibodies employed in the table attached at the end of this document. For the quantification of c-Fos immunostaining, all samples were processed for immunostaining in parallel, imaged in a Nikon Eclipse 90i microscope and analyzed using automated measurement tool in the NIS-Element software, version 4.51.

References

1. Ritchie ME, Phipson B, Wu D, Hu Y, Law CW, Shi W, Smyth GK. Limma powers differential expression analyses for RNA-sequencing and microarray studies. *Nucleic Acids Res* **2015**;43(7):e47.
2. Song, A. S., Najjar, A. M., & Diller, K. R. Thermally Induced Apoptosis, Necrosis, and Heat Shock Protein Expression in Three-Dimensional Culture. Journal of biomechanical engineering **2014**;136(7):071006.
3. Garaulet G, Alfranca A, Torrente M, Escolano A, López-Fontal R, Hortelano S, Redondo JM, Rodríguez A. IL10 released by a new inflammation-regulated lentiviral system efficiently attenuates zymosan-induced arthritis. *Mol Ther* **2013**; 21(1):119-130.

List of antibodies.

Antibody	Reference	Provider
Col2a1	ab85266	AbCam
S100	Z0311	Dako
c-Fos	Sc7202	Santa Cruz Biotech
c-Jun	610326	BD Pharmingen
polyclonal donkey anti-goat	705-035-147	Jackson
CD105	105F-100T	Immunostep
CD44	15-0441-83	eBioscience
CD29	11-0297-73	eBioscience
CD73	561254	BD Pharmingen
CD90	555595	BD Pharmingen
CD45	11-0459-73	eBioscience
Vimentin	Ab8069	AbCam
BrDU	347580	BD Pharmingen
MPM-2	16-220	Millipore
Cyclin-A1	SC-271682	Santa Cruz Biotech
Anti-mitochondria	ab3298	AbCam
Osteocalcin	ab76690	AbCam
Osteonectin	MAB9401	R&D
SMA	A5228	Sigma
Ki67	RM-9106-R7	Thermo Scientific
GFP	A11122	Invitrogen
AnnexinV-PE	BMS306PE/100	eBiosciences

List of primers used in qRT-PCR analysis.

Primers	Sequence	T (°C)
FOS-F	CCGACTCCTTCTCCAGCAT	58
FOS-R	TCACCGTGGGGATAAAGTTG	
RUNX2-F	CGGCCCTCCCTGAACTCT	58
RUNX2-R	TGCCTGCCTGGGGTCTGTA	
SOX9-F	GACTTCCGCGACGTGGAC	60
SOX9-R	GTTGGGCGGCAGGTACTG	
CCNA1-F	GCACCCTGCTCGTCACTTG	60
CCNA1-R	CAGCCCCCAATAAAAGATCCA	
MYBL2-F	CTGTACCGATGGGCTCCTGTT	60
MYBL2-R	TGCCAGGGAGGACAGACAAT	
TPM1-F	CTCTCAACGATATGACTTCCA	58
TPM1-R	TTTTTTTAGCTTACACAGTGTT	
GAPDH-F	GCCAAGGTCATCCATGACAACT	58
GAPDH-R	AGGGCCATCCACAGTCTTCTG	

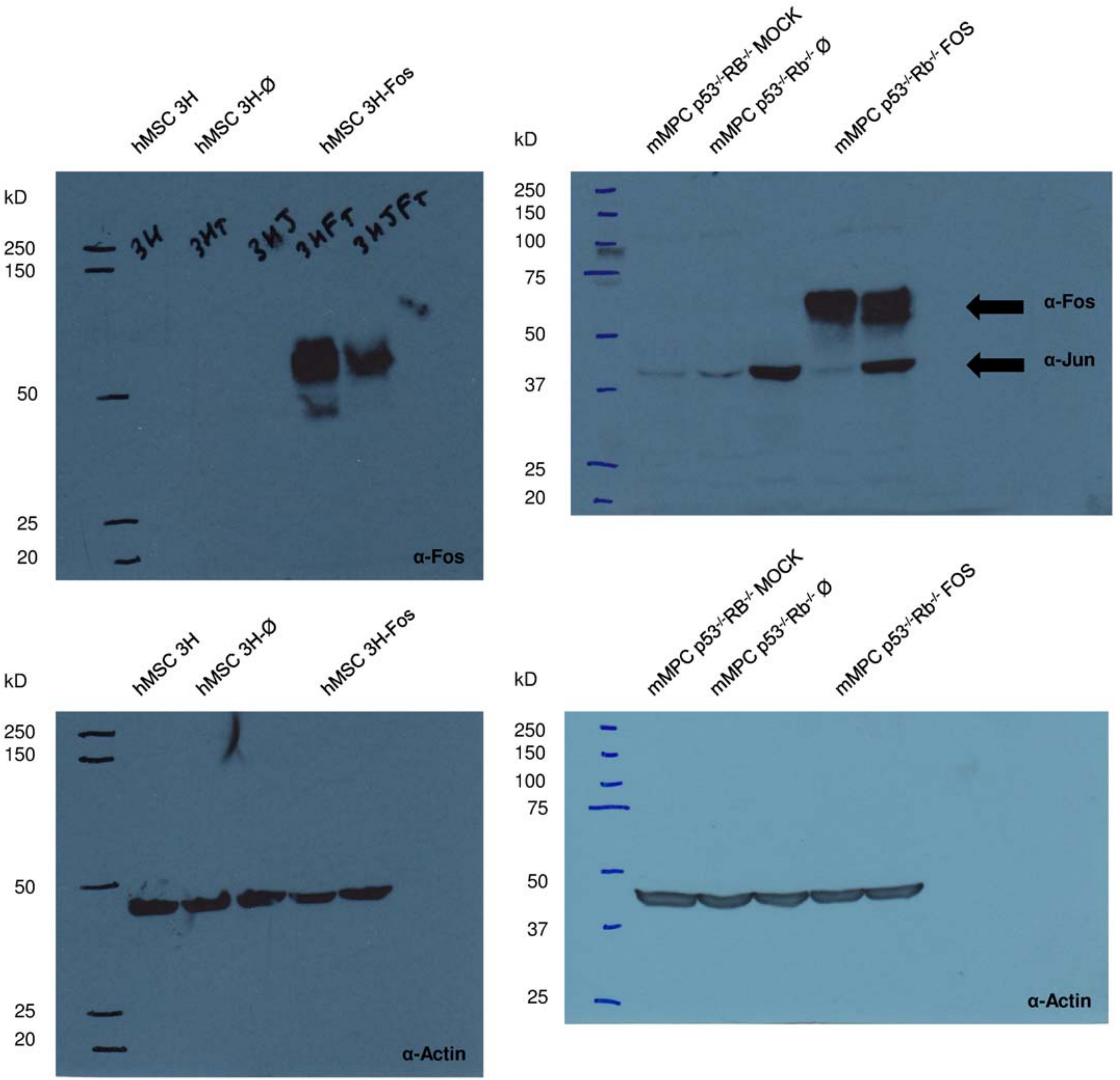

hMSC 3H
hMSC 3H-Ø
hMSC 3H-Fos
kD
250
150
50
25
20
3H
3HT
3HJ
3HFT
3HJFT
α-Fos
mMPC p53-/-RB-/- MOCK
mMPC p53-/-Rb-/- Ø
mMPC p53-/-Rb-/- FOS
kD
250
150
100
75
50
37
25
20
α-Fos
α-Jun
hMSC 3H
hMSC 3H-Ø
hMSC 3H-Fos
kD
250
150
50
25
20
α-Actin
mMPC p53-/-RB-/- MOCK
mMPC p53-/-Rb-/- Ø
mMPC p53-/-Rb-/- FOS
kD
250
150
100
75
50
37
25
α-Actin
